# Fate Specification of Neural Plate Border by Canonical Wnt Signaling and *Grhl3* is Crucial for Neural Tube Closure

**DOI:** 10.1016/j.ebiom.2015.04.012

**Published:** 2015-04-18

**Authors:** Chiharu Kimura-Yoshida, Kyoko Mochida, Kristina Ellwanger, Christof Niehrs, Isao Matsuo

**Affiliations:** a Department of Molecular Embryology, Osaka Medical Center, Research Institute for Maternal and Child Health, Osaka Prefectural Hospital Organization, 840 Murodo-cho, Izumi, Osaka 594-1101, Japan; b Division of Molecular Embryology, DKFZ-ZMBH Alliance, 69120 Heidelberg, Germany; c Institute of Molecular Biology, 55128 Mainz, Germany

**Keywords:** Surface ectoderm, Neural tube closure, Neural fold, Uncommitted progenitors, Grainyhead-like family, Canonical Wnt, Wnt antagonists, Neural tube defects

## Abstract

During primary neurulation, the separation of a single-layered ectodermal sheet into the surface ectoderm (SE) and neural tube specifies SE and neural ectoderm (NE) cell fates. The mechanisms underlying fate specification in conjunction with neural tube closure are poorly understood. Here, by comparing expression profiles between SE and NE lineages, we observed that uncommitted progenitor cells, expressing stem cell markers, are present in the neural plate border/neural fold prior to neural tube closure. Our results also demonstrated that canonical Wnt and its antagonists, DKK1/KREMEN1, progressively specify these progenitors into SE or NE fates in accord with the progress of neural tube closure. Additionally, SE specification of the neural plate border via canonical Wnt signaling is directed by the *grainyhead-like 3 (Grhl3*) transcription factor. Thus, we propose that the fate specification of uncommitted progenitors in the neural plate border by canonical Wnt signaling and its downstream effector *Grhl3* is crucial for neural tube closure. This study implicates that failure in critical genetic factors controlling fate specification of progenitor cells in the neural plate border/neural fold coordinated with neural tube closure may be potential causes of human neural tube defects.

## Highlights

• Neural plate border/neural fold possesses stem cell-like characters during primary neurulation.
• Canonical Wnt and its antagonists progressively specify progenitors into surface or neural fates upon neural tube closure.
• Fate specification into surface ectoderm in the neural fold is directed by the *Grhl3* transcription factor.
• Fate specification of uncommitted progenitors in the neural plate border is intimately coupled to neural tube closure.

## 1 Introduction

Neural tube defects are severe developmental disorders of the brain or spinal cord and one of the most common with a frequency of about 1 per 1000 births in humans (Mitchell, 2005). It is still unclear how genetic and environmental factors are associated with human neural tube defects. Neural tube defects arise when the neural tube fails to close completely during early embryonic development. Neural tube closure proceeds progressively at different levels of the anterior–posterior axis during primary neurulation in which the neural plate bends, folds up and fuses along the dorsal midline of neural folds.

Notably, neural ectoderm (NE) and surface ectoderm (SE) initially adjoin within an ectodermal sheet prior to neural tube closure and thereafter the sheet completely separates the two fates by the end of neural tube closure. Thus, it can be assumed that fate specification within the ectodermal sheet into NE or SE should be precisely controlled in conjunction with neural tube closure in both time and space. Indeed, genetic clonal analyses of cell lineage segregations in the mouse embryo have suggested that ectodermal progenitor cells, which are not committed to the NE or SE, are present during early to mid-gastrulation (Tzouanacou et al., 2009, Lawson et al., 1991). However, little is known about what, when, and how molecular mechanisms control the fate specification decision between NE and SE cells in the neural plate border/neural fold, where these cells adjoin.

Moreover, it is still unknown whether uncommitted progenitor cells remain in the neural plate border following NE and SE specification during neurulation in vertebrate embryos. Indeed, much attention has been paid to the neural plate border between the NE and SE in light of the major source of the neural crest and placodes, and for decades many studies have explored the mechanisms underlying neural crest specification in the neural plate border (Patthey and Gunhaga, 2011, Sauka-Spengler and Bronner-Fraser, 2008, Grocott et al., 2011, Steventon et al., 2014, Stuhlmiller and Garcia-Castro, 2012, Weston and Thiery, 2015).

The present study reveals that the neural plate border possesses an uncommitted progenitor state prior to the midline fusion of neural folds during neural tube closure. Our data provide evidence that canonical Wnt signaling promotes SE specification, whereas the Wnt antagonist, *Dickkopf1* (*Dkk1*), induces NE specification in the neural plate border. Additionally, *Grainyhead-like 3* (*Grhl3*) transcription factor, which is essential for neural tube closure, specifies the SE cell fate under the control of canonical Wnt. Thus, we propose that fate specification of the neural plate border/neural fold via canonical Wnt signaling and the *Grhl3* factor is crucial for neural tube closure. This study implicates that the canonical Wnt pathway may have an important role in human neural tube closure. Moreover, failure in critical factors controlling fate specification of uncommitted progenitor cells in the neural fold coordinated with neural tube closure may induce human NTDs.

## 2 Materials and Methods

### 2.1 Constructions of transgene vectors and generation of transgenic mice

Mouse *Dkk1*, *Wnt8A* and *Grhl2* cDNA fused to the *CAG* promoter was ligated to the *LacZ* gene flanked by two *loxP* sequences, as described previously (Kimura-Yoshida et al., 2005). Mouse *Grhl2* and *Grhl3* cDNAs were obtained from EMAGE clone #6306106 and #30607679, respectively. These cDNAs were inserted into the *CAG* cassettes. These constructs were microinjected into the pro-nucleus of the fertilized eggs from CD-1 as described (Nagy et al., 2003)

### 2.2 Mouse Genotyping

Genotyping of *CAG-Dkk1* transgenic mice obtained using the PCR primers *CAG-pro* (5′-TAGAGCCTCTGCTAACCATGTTCATGCCTT-3′) and *CAG-Dkk1* (5′-TCAGCGCAAGGGTAGGGCTGGTAGTTGTCA-3′), yielding 443 bp. *CAG-Wnt8A* transgenic mice were genotyped as described (Kimura-Yoshida et al., 2005). *Dkk1* null mutant mice were identified with three primers: *mdkk1-5*′ (5′-GCAACGCTAAAGGGTTAATCAGCAACTATA-3′), *mdkk1-frt5*′ (5′-CCCCGGGAACTACTGCAAAAATGGT-3′), and *mdkk1-frt3*′(5′-GTGCTCAAACACAAGCCAGTGACGA-3′), yielding 180 bp as the wild-type allele and 400 bp as the mutant allele. *Grhl3-Cre-IRES-nlsLacZ* knock-in mice were obtained from the Mutant Mouse Regional Resource Center (MMRRC) and genotyped with the following primers: *Grhl3-cre* (5′-AATTAAGAGACGAGTGGTCAGCAGCGCCTG-3′), *Grhl3-cre wt rev* (5′-ACCCTTACAAATTGCCGTGTGAATCCGGGC-3′), and *Grhl3-cre mut* (5′-GCAGCCCGGACCGACGATGAAGCATGTTTA-3′), yielding 213 bp as the wild-type allele and 370 bp as the mutant allele.

*β-Catenin* knockout mice were genotyped as described (Kimura-Yoshida et al., 2005). The *CAG-Grhl2* allele was genotyped by PCR of genomic DNA using the following primers: *CAG-pro* primer (described aforementioned) and *Grhl2-rev1* 5′-CAGATATGACTTCCAGGCCTCATCCTCACT-3′, yielding 330 bp as a PCR fragment. Rosa GNZ knock-in mice (*Gt(Rosa)26Sor^tm1Joe/J^*) were obtained from the Jackson Laboratory (Stoller et al., 2008). Rosa GNZ mutant mice were identified with these primers: forward 5′-CTCCCAAAGTCGCTCTGAGTTGTTATCAGT-3′, reverse 5′-CAACGCCCACACACCAGGTTAGCCTTTAAG-3′, yielding 265 bp as the wild-type allele and forward 5′-TCGCTACCATTACCAGTTGGTCTGGTGTCA-3′ 443 bp as the mutant allele.

### 2.3 DNA Microarray

Wild-type embryos were dissected in PBS with calcium and magnesium. The embryos were then incubated in pancreatin/trypsin in calcium- and magnesium-free PBS for dissection. The surface ectoderm and neural ectoderm at the forebrain level, which does not include cephalic mesenchymal cells, were isolated using tungsten needles. The numbers of embryos used for three independent biological replicate experiments were as follows: replicate 1, surface ectoderm 1 (SE1) n = 109, neural ectoderm 1 (NE1) n = 46; replicate 2, SE2 n = 117, NE2 n = 43; and replicate 3, SE3 n = 114, NE3 n = 45. Isolated tissues were stored at − 80 °C after being soaked in RNA*later* (Applied Biosystems). Collected tissues were purified using a TRIzol-plus purification kit (Invitrogen, cat. no. 12183-555). The quality of purified RNA samples was confirmed using Experion (BioRad). cRNA was prepared using the illumine Total Prep RNA Amplification Kit (Illumina). Samples were hybridized to a MouseWG6-v2 array (Illumina) and scanned with a BeadArray Reader (Illumina). Raw data were analyzed using Bead Studio software (Illumina), and pairwise comparisons were done between the SE and NE populations for each of the three biological replicates. We noted that there was good correspondence between replicated genes with a two-fold change. The accession number for the microarray data reported in this paper is GSE67977.

### 2.4 TEM and SEM

TEM and SEM procedures for mouse embryos followed techniques previous described (Kimura-Yoshida et al., 2005, Kimura et al., 2000).

### 2.5 Whole Embryonic Culture

Wild-type and *CAG-Dkk1* embryos at the 4- to 5-somite stage were cultured in DMEM supplemented with 20% fetal bovine serum and 1 × nonessential amino acid in a 5%CO_2_ incubator at 37 °C.

### 2.6 Immunohistochemistry

Mouse embryos were dissected and embedded in OCT and frozen overnight at − 80 °C. Cryosections were cut at 10 μm using a Microm HM500 OM cryostat, mounted onto superfrost slides, and stored at − 80 °C until use. After thawing, the slides were fixed in 4% paraformaldehyde in PBS and washed in 0.1% TritonX in PBS (PBT). The sections were then incubated in blocking solution (1% BSA/10% goat serum/PBT) for 1 h. Incubation of the primary antibody diluted in blocking solution was performed overnight, as shown in Fig. S9. Sections were then washed three times in PBT for 5 min per wash and incubated for 2 h at RT with the second antibody in blocking solution. The sections were further washed three times with PBT for 5 min per wash and placed onto slides using a mounting medium. Immunohistochemistry for whole-mount embryos and paraffin embedding sections were performed as described (Shimokawa et al., 2011).

### 2.7 Histology, electron microscopy, X-gal staining, and in situ hybridization

For the histological analysis, embryos were fixed in Bouin's fixative, dehydrated, and embedded in paraplast. Serial sections (thickness, 7 μm) were generated and stained with hematoxylin and eosin (HE). X-gal staining and *in situ* hybridization were performed as described (Kimura-Yoshida et al., 2005, Kimura et al., 1997, Kimura et al., 2000). The following template cDNA plasmids were used for the synthesis of the probes: *Foxi2* (nt 1554–2267), *Grhl2* (nt 203–603), *Grhl3* (nt 207–693), *Klf5* (nt 986–1590), *Kremen1* (nt 3796–4626), *Lrp4* (nt 7220–7720), *Lrp5* (nt 2888–3444), *Lrp6* (nt 2941–3446), *Lrp10* (nt 2542–2941), *Twist1* (nt 666–1630), and *Wnt6* (IMAGE clones 6511061).

### 2.8 Ethics Statement

All mouse studies followed the fundamental guidelines for proper conduct of animal experiments and related activities in academic research institutions under the jurisdiction of the Ministry of Education, Culture, Sports, Science, and Technology of Japan and were approved by institutional committees at the Osaka Medical Center and Research Institute for Maternal and Child Health for animal and recombinant DNA experiments.

## 3 Results

### 3.1 Uncommitted Progenitor Cells are Present in the Neural Plate Border Prior to Neural Fold Fusion

**Fig. 1:**
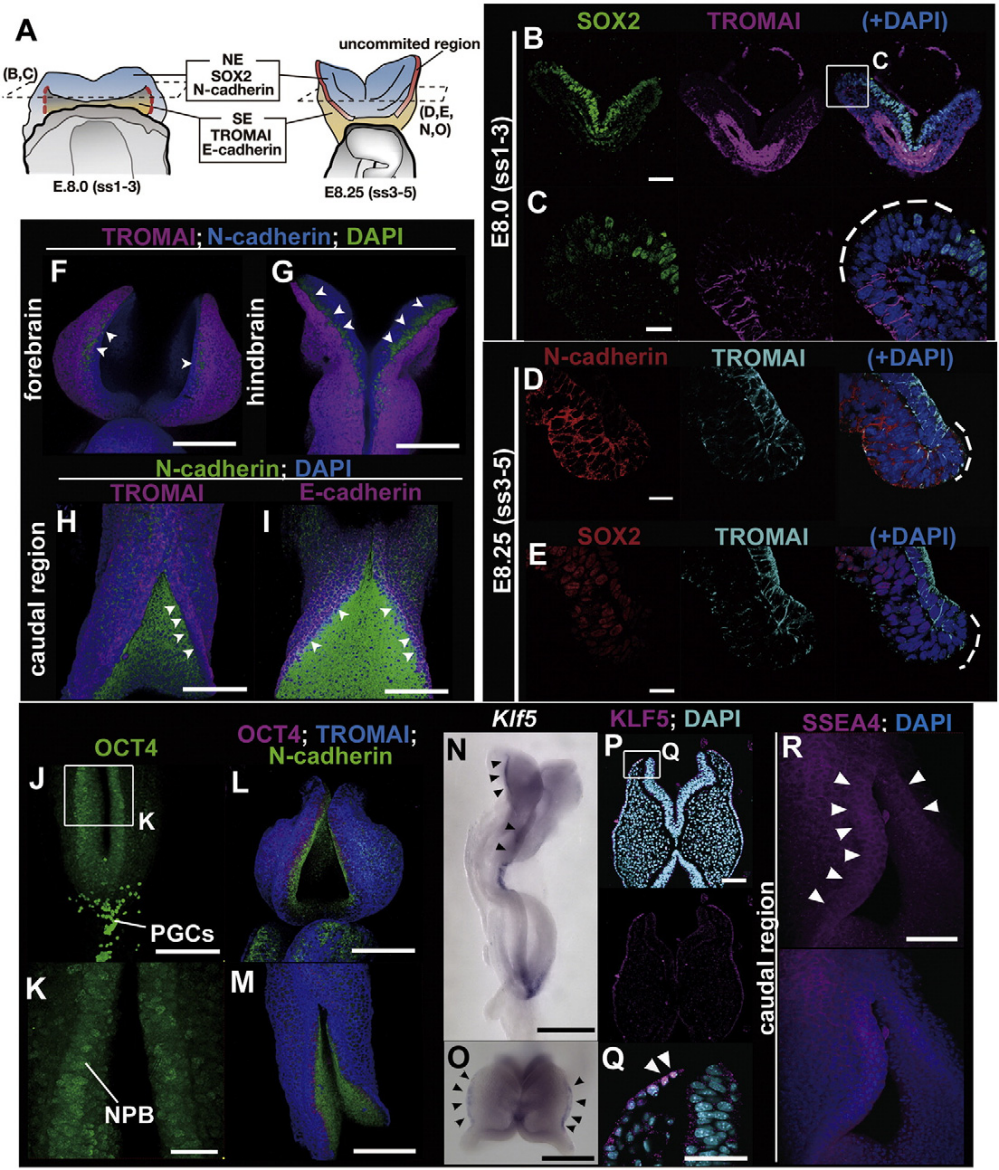
Comparative expression analysis with NE, SE, and stem cell markers during primary neurulation. (A) Schematic expression patterns in E8.0 (somite stages 1–3) and E8.25 (somite stages 3–5) embryos. (B–M, P–R) Images of section (B–E, P, Q) and whole-mount (F–M, R) immunohistochemistry stained at E8.0 (B, C), E8.25 (D, E) and E8.5 (somite stages 5–8) (F–I, J–M, R) with Sox2/TROMAI (B, C, E), N-cadherin/TROMAI (D, F–H), N-cadherin/E-cadherin (I), OCT4 (J, K), OCT4/TROMAI/N-cadherin (L, M) KLF5 (P, Q) and SSEA4 (R). (N, O) Whole mount *in situ* hybridization using *Klf5* probe at E8.5 (dorsolateral view, N; dorsal view, O). Each panel shows a region where neither SE nor NE markers are expressed as dotted lines (C–E) and arrowheads (F–I). The expression of OCT4, a stem marker, specifically labels the boundary between NE and SE at E8.5, although the expression level is lower than that of primordial germ cells (J–M). *Klf5*, which is a *Krüppel-like* transcription factor that plays crucial roles in maintaining embryonic stem (ES) cell pluripotency, is expressed in the neural plate border (arrowheads, N–Q). SSEA4 is a cell surface marker of human ES, embryonic germ (EG), and induced pluripotent stem (iPS) cells and is expressed in the proximity of the neural plate border (arrowheads, R). NPB, neural plate border; PGCs, primordial germ cells. Scale bar represents 300 μm in N; 200 μm in F–J, L, M; 100 μm in B, O, P, R; 50 μm in K, Q; and 20 μm in C–E.

During primary neurulation, NE and SE cells adjoin directly within the same ectodermal sheet, and then the neural plate separates from the SE during the completion of neural tube closure. First, for the determination of how and when the cell fate is specified in conjunction with epithelial fusion during neural tube closure, the expressions of several NE and SE markers were analyzed (Fig. 1). We found that NE markers such as N-cadherin and SOX2 and SE markers such as TROMAI/Keratin8 and E-cadherin are expressed roughly in their respective regions during early neurulation, between E8.0 and E8.5 (somite stages [ss] 1–3 and 5–8, respectively; Fig. 1A–I). However, the uncommitted progenitors between SE and NE in the neural plate border/neural fold — where neither SE nor NE is specified — are present until the completion of epithelial fusion (Fig. 1A–I; dotted lines in C–E, arrowheads in F–I).

For the molecular characterization of these uncommitted progenitor cells, the expressions of several stem cell markers were examined (Fig. 1J–R). OCT-3/4 expression, a stem cell marker, was detected in the neural plate border/neural fold, although at a lower level than that in primordial germ cells (Fig. 1J–M). Two additional stem cell markers, *Klf5* transcripts and its proteins and SSEA-4, were also expressed in the neural plate border (Fig. 1N–R, arrowheads). These findings indicate that a neural plate border region during neurulation possesses a stem cell-like character.

**Fig. 2:**
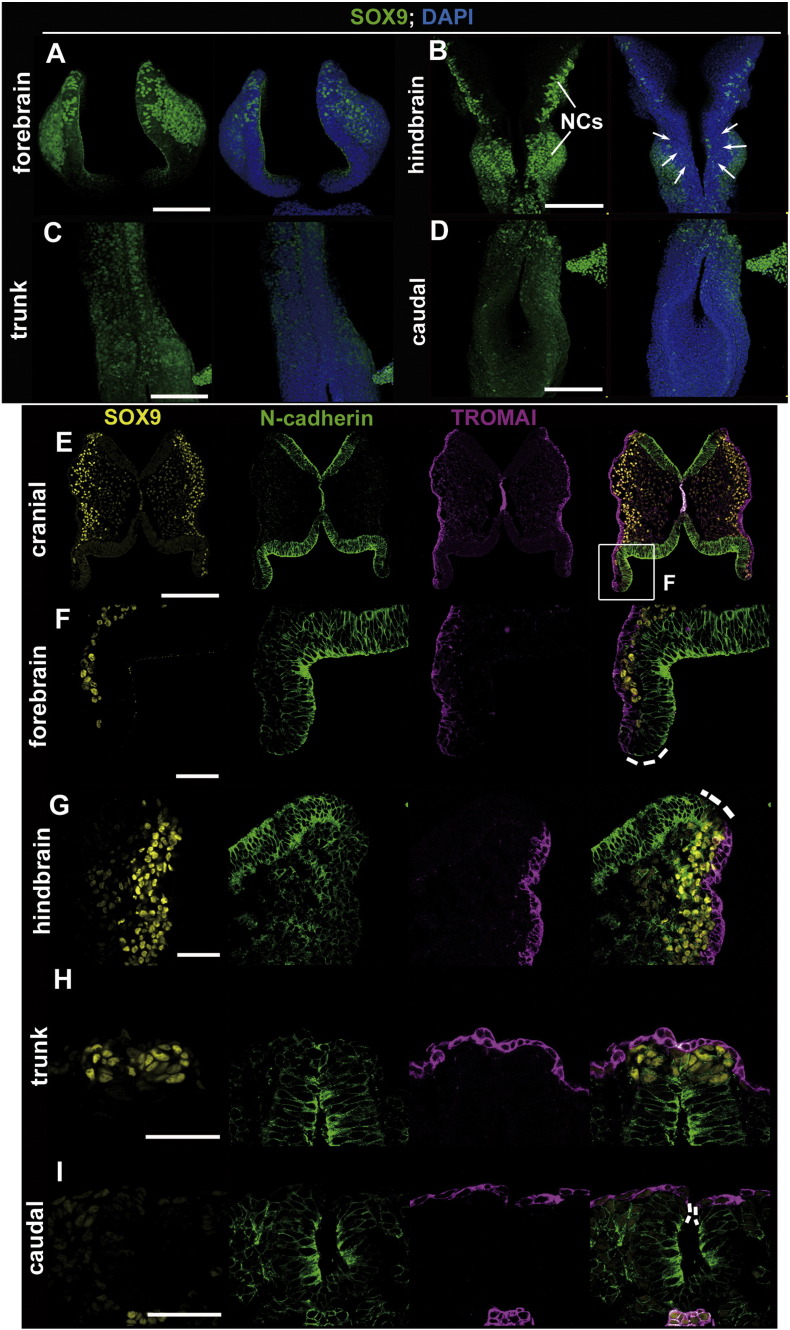
Behaviors of SOX9-positive neural crest cells during neurulation. (A–D) Whole-mount immunohistochemistry stained at E8.5 with SOX9 antibody. SOX9-positive cells are present in pre- and post-migratory neural crest cells at the forebrain, hindbrain and trunk levels but few were present at the caudal level (A–D). In this study, we defined “trunk” as the anterior spinal cord level in which the neural tube is closed completely, and “caudal” as the caudal spinal cord level in which the neural tube is still open. (E–I) Sectional images of immunohistochemistry at the level of the forebrain (E, F), hindbrain (E, G), trunk (H) and caudal (I) with SOX9 (yellow)/N-cadherin (green)/TROMAI (magenta). At the level of the hindbrain, SOX9-positive cells were present in the proximity of the neural plate border (G; white dotted lines). Dotted white lines indicate the neural plate border. NCs, neural crest cells. Scale bar represents 200 μm in A–E; 50 μm in F–I.

To further assess whether these neural plate border progenitors are relevant to the population of the neural crest, we analyzed the expression of SOX9, a pre-migratory and post-migratory neural crest marker (Fig. 2). A very few SOX9-positive cells were located in the neural plate border at the forebrain (Fig. 2A, E, F). At the hindbrain level, a considerable amount of SOX9-positive cells were delaminated from in the vicinity of the neural plate border (Fig. 2B, G). However, SOX9-negative cells appeared to be present and still remained at an uncommitted state at this stage at both the forebrain and hindbrain levels (Fig. 2F, G, dotted lines). Additionally, given that SOX9-positive cells were localized in the dorsal neural tube at the trunk level and undetectable at the caudal level, neural crest cells are not directly from the uncommitted progenitors in the neural plate border during neurulation at the trunk and caudal levels (Fig. 2C, D, H, I).

These findings indicate that although uncommitted progenitor cells in the neural plate border contributed to neural crest lineages to some extent (at least at the level of the hindbrain), the decision of fate specification to the neural crest appears to be not intimately linked to neural tube closure. Taken together, the fate specification to SE or NE in the neural plate border appears to be intimately coupled to neural tube closure.

### 3.2 Wnt Signaling Modulators DKK1 and KREMEN1 are Localized at the Neural Plate Border

To identify what types of signaling pathways are involved in the fate specification between SE and NE cells during neurulation, we carefully separated the SE and NE from the forebrain level of mouse embryos at E8.25–E8.5 (pooled samples from 3- to 5-somite stage embryos; three biological replicates) and compared the whole genome-wide gene expression profiles by means of DNA microarray (Fig. S1). Initially, we surveyed the Wnt, FGF, and BMP ligands, which are thought to be involved in the specification between neural and epidermis fates prior to or during gastrulation in other vertebrates (Murry and Keller, 2008).

**Fig. 3:**
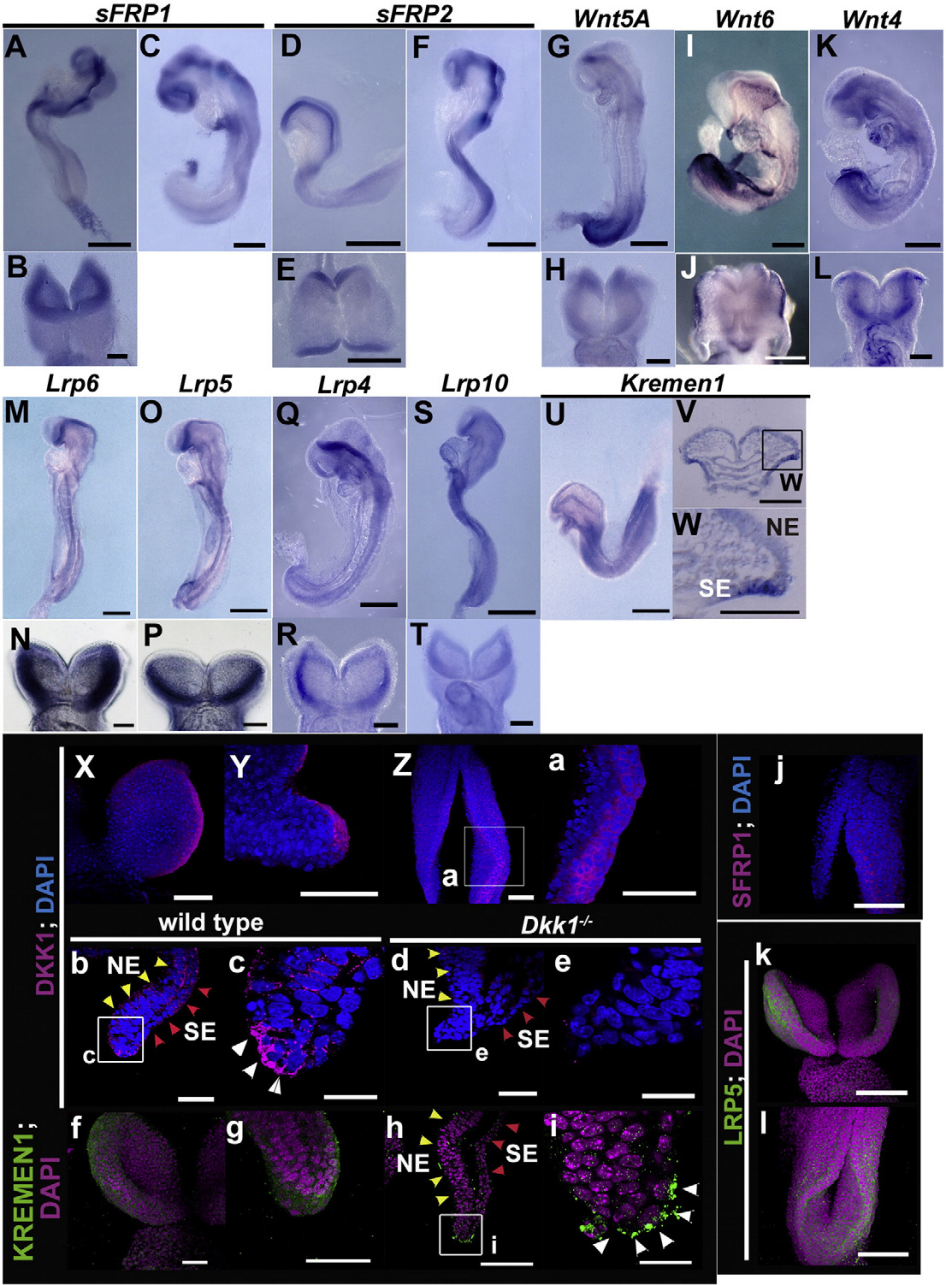
Wnt signaling-related genes are expressed in the proximity of the neural plate border. (A–W) Whole-mount *in situ* hybridization analysis (A–U) and its sectional views (V, W) of wild-type embryos at E8.25 (D, E, U–W), E8.5 (A, B, F, M–P, S, T) and E8.75 (C, G–L, Q, R). Lateral views (A, C, D, F, G, I, K, M, O, Q, S, U), frontal views (B, H, L, N, P, R, T), and dorsal view (E, J). *sFRP1* (A–C), *sFRP2* (D–F), *Wnt5A* (G, H), *Wnt6* (I, J), *Wnt4* (K, L), *Lrp6* (M, N), *Lrp5* (O, P), *Lrp4* (Q, R), *Lrp10* (S, T) and *Kremen1* (U–W). (X-l) Immunohistochemical analyses of whole-mount embryos (X-a, f, g, j–l) and transverse sections (b–e, h, i) in wild-type (X-c, f–l) and *Dkk1^−/−^* embryos (d, e). DKK1 (X-e; magenta), KREMEN1 (f–i; green), SFRP1 (j; magenta) and LRP5 (k, l; green) proteins are localized at the neural plate border (white arrowheads in c and i), and DKK1 expression is not detected in the *Dkk1^−/−^* embryos (d, e). NE, neural ectoderm; SE, surface ectoderm. Scale bars: 300 μm in A, C, D, F, G, I, K, M, O, Q, S, U; 200 μm in j–l; 100 μm in B, E, H, J, L, N, P, R, T, V-a, f–h; 50 μm in b, d, e; 20 μm in c, i.

The expression profiles of signal transduction-related genes showed that the expression of Wnt-related molecules is upregulated or downregulated between SE and NE, apparently depending on their functions (Fig. S1). In contrast, the expression of other ligands, BMP and FGF, was observed exclusively in the SE and NE, respectively. The subsequent expression analysis with whole-mount in situ hybridization indicated that Wnt ligands, co-receptors, and receptors were actually expressed in the proximity of the neural plate border between the SE and NE (Fig. 3A–W).

To explore the roles of Wnt signaling in fate specification, we examined the protein expressions of DKK1 and KREMEN1, which are antagonists/modulators for canonical and noncanonical Wnt (Glinka et al., 1998), more precisely (Fig. 3X-i). DKK1 proteins were detected in the neural plate border at both the cranial and caudal levels during neurulation (Fig. 3X-c; arrowheads). This local DKK1 expression was not observed in the *Dkk1* knockout mouse embryo (Fig. 3d, e). Concurrently, KREMEN1 proteins as well as its transcripts were localized specifically at the neural plate border (Fig. 3U–W, f–i; arrowheads). Similarly, SFRP1 and LRP5 proteins, an antagonist and co-receptor of canonical Wnt, respectively, were also prominently localized in the neural plate border (Fig. 3j–l). Together, these findings suggested that Wnt signaling is one of the candidate pathways controlling the specification between NE or SE fate in uncommitted progenitors during neural tube closure.

### 3.3 Canonical Wnt Signaling Promotes the SE Fate in the Neural Plate Border During Primary Neurulation

**Fig. 4:**
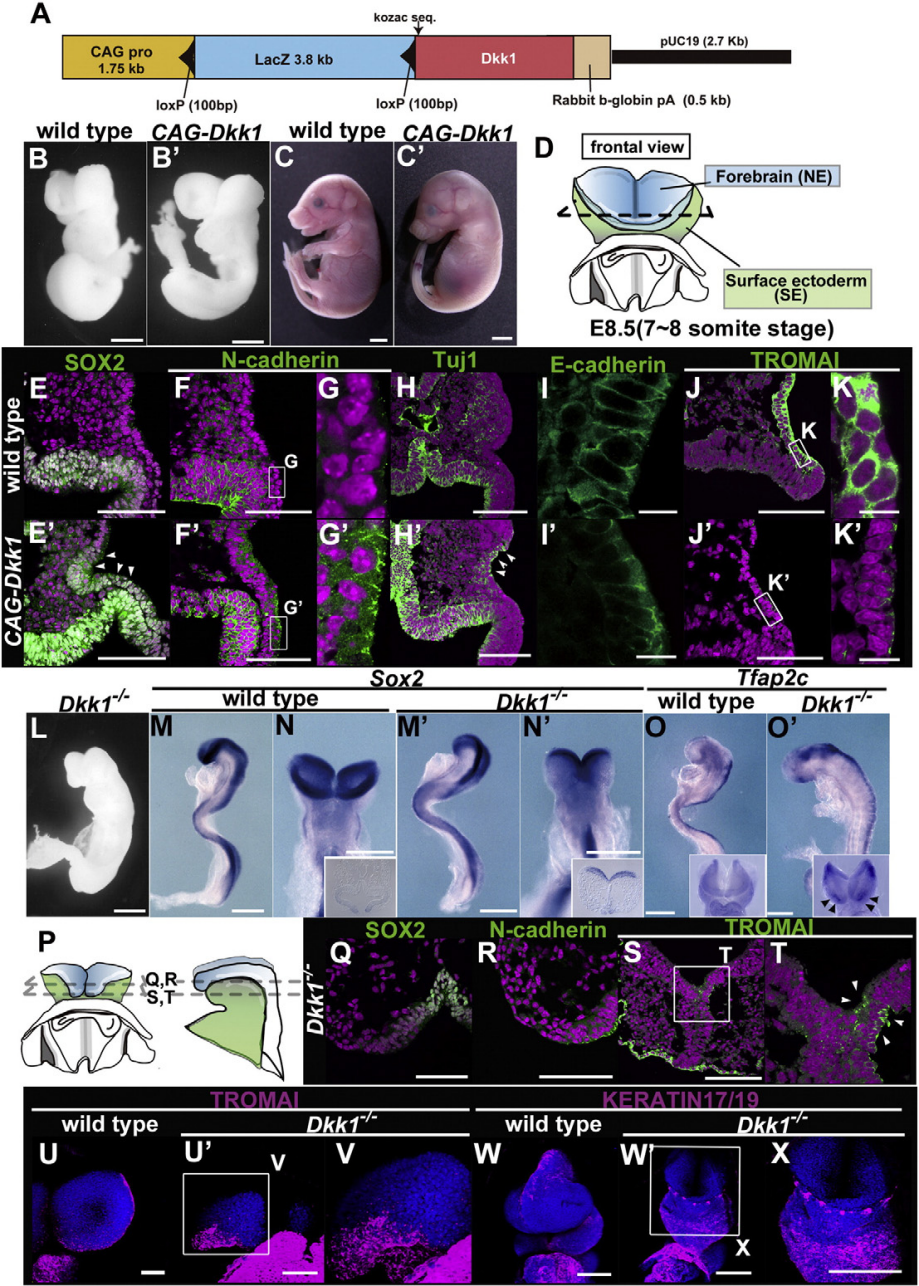
Molecular marker analyses in *CAG-Dkk1* and *Dkk1^−/−^* mutant embryos. (A) A schematic construct of the *CAG-Dkk1* transgene. (B–C′, L) The gross appearances of wild-type (B, C), *CAG-Dkk1* (B′, C′), and *Dkk1^−/−^* (L) embryos at E8.5 (B, B′, L) and E18.5 (C, C′). (D, P) Transverse sections of embryos. Each section plane is represented by a corresponding line in the scheme in *CAG-Dkk1* (D) and *Dkk1^−/−^* (P). (E–K′, Q–X) Immunohistochemical analyses with frozen sections of the wild-type (E–K), *CAG-Dkk1* (E′–K′), and *Dkk1^−/−^* (Q–T) and with whole embryos of the wild-type (U, W) and *Dkk1^−/−^* embryos (U′, V, W′, X) at E8.5 (E–K′, Q–T), E8.25 (U–V) and E8.75 (W–X). SOX2 (E, E′, Q), N-cadherin (F–G′, R), Tuj1 (H, H′), E-cadherin (I, I′), TROMAI (J–K′, S–V), and KERATIN17/19 (W, X) merged with nuclei staining (TOTO-3; magenta in E-H′, J–K′, Q–T; blue in U–X). (M–O′) Whole-mount *in situ* hybridization in the wild-type (M–O) and *Dkk1^−/−^* embryos (M′–O′) at E8.5. Lateral and frontal views of *Sox2* expression (M–N′) with sectional views and lateral views of *Tfap2c* expression with their frontal views (O, O′). Scale bars: 3 mm in C, C′; 300 μm in B, B′, L–O′, W–X; 100 μm in E–F′, H, H′, J, J′, Q–U′; 10 μm in I, I′, K, K′.

To determine whether Wnt signaling affects these two cell fates, we generated transgenic mice misexpressing *Dkk1* (Fig. 4A). To create *Dkk1*-misexpressing embryos, we crossed the *CAG-loxP-lacZ-loxP-Dkk1* transgenic mouse — in which the *lacZ* gene is flanked by two *loxP* sites and the *Dkk1* cDNA is misexpressed under the control of the *CAG* promoter — with the *β-actin-cre* transgenic mouse (Fig. 4A). The resultant double-transgenic embryos expressed the Dkk1 protein ectopically (*CAG-Dkk1*) (Kimura-Yoshida et al., 2005). Despite rather ubiquitous *Dkk1* misexpression, given that the inhibitory effect on Wnt signaling through *Dkk1* appears to require Kremen1/2 co-receptors and Lrp5/6 receptors (Cruciat and Niehrs, 2013), *Dkk1* will attenuate Wnt signaling notably in local areas where Kremen1/2 and Lrp5/6 as well as Wnt ligands are expressed during neurulation (Fig. 3).

Morphological abnormalities in *CAG-Dkk1* embryos were detectable around E8.5. Notably, the forebrain appeared to be expanded (Fig. 4B′) and the growth of the forelimbs and hindlimbs was greatly diminished in *CAG-Dkk1* embryos at E18.5 (Fig. 4C′). Considering the effects of *Dkk1* deficiency, which exhibits ectopic digits and forebrain reduction (Mukhopadhyay et al., 2001), the *CAG-Dkk1* morphological deformities also support the notion that the effect of *Dkk1* misexpression is the inverse of the effect of *Dkk1* deficiency. Concurrently, the expression patterns of the canonical Wnt markers β-catenin and Top-gal reporter were down-regulated in *Dkk1*-misexpressed embryos (Fig. S2A-F, A′–F′).

To explore the possibility that *Dkk1* can affect the specification between NE- and SE cell fates, we examined molecular markers in *CAG-Dkk1* embryos with high-resolution immunohistochemistry (Fig. 4D–K′). The NE markers SOX2, N-cadherin, and Tuj1 were localized in the NE as they are normally (Fig. 4E–H), but they were also found ectopically in the presumptive SE region in the *CAG-Dkk1* embryos (Fig. 4E′–H′; arrowheads). Conversely, the SE markers E-cadherin and TROMAI were severely reduced in the SE region of the *CAG-Dkk1* embryos (Fig. 4I′–K′). Together these findings suggested that *Dkk1* misexpression promotes the specification of NE fate and inhibits that of SE fate in the neural plate border.

Consistent with the above molecular markers, the presumptive SE of *CAG-Dkk1* embryos was transformed from a simple epithelium to a pseudostratified- or stratified-like epithelium (Fig. S3A–C′). To analyze the histological abnormalities of SE and NE in *CAG-Dkk1* embryos more precisely, we examined their fine structures by means of conventional scanning electron microscopy (SEM) and by viewing ultra-thin sections using transmission electron microscopy (TEM) (Fig. S3D–H′). In the wild-type embryos at E8.5, the morphological features of the SE, neural plate border and NE regions were distinct, and the neural plate border, previously referred to as “flattened cells” extended to the end of neural folds along with the longitudinal axis (Fig. S3D, E; flattened border cells in blue, NE in pink and SE in yellow, respectively) (Waterman, 1976). In contrast, flattened cells in the neural plate border between the NE and SE of the *CAG-Dkk1* embryos became unclear, and the morphological features of the border region were more similar to those of the NE (Fig. S3D′–H′; the border region in green). Together, the fine morphological analyses suggested that in the *CAG-Dkk1* embryos, the medial SE region, which is derived from the neural plate border, possessed the characteristics of NE.

In addition, our developmental studies of the *CAG-Dkk1* embryos using an *in vitro* culture system indicated that the timing of the neural tube closure appeared to be delayed in the *CAG-Dkk1* embryos (Fig. S3I–K′). The telencephalic neuropore, which closes nearly within 6 h in the wild type (ss 3–4; Fig. S3I–K), was still open and extended laterally after 6 h of culture in the *CAG-Dkk1* embryos (Fig. S3I′–K′). This finding suggests the possibility that altered cell fate specification of the neural plate border in *CAG-Dkk1* embryos can affect the timing of the neural tube closure.

To investigate whether *Dkk1* deficiency may have the opposite effect on the specification between SE and NE, we analyzed *Dkk1* null mutant embryos with specific markers (Fig. 4L–X, Fig. S2D′′–F′′) (Pietila et al., 2011). The NE markers SOX2 and N-cadherin were significantly reduced in the *Dkk1*^−/−^ embryos at E8.5 (Fig. 4M′, N′, Q, R). Conversely, the SE markers *Tfap2c* and TROMAI were upregulated in the *Dkk1*^−/−^ embryos, and they were also ectopically found in the presumptive NE region at both E8.25 and E8.5 during neurulation (Fig. 4O′, S, T, U′, V, W′, X; arrowheads). These findings demonstrate that the anterior neural plate border in *Dkk1*-deficient embryos was specified into an SE cell fate at the expense of an NE cell fate, and that these are the inverse of the ectodermal phenotypes in *Dkk1*-misexpressing embryos (Fig. 4E′–K′).

To determine whether these two cell fates can be affected by canonical Wnt, we generated transgenic mice in which *Wnt8A*, a canonical Wnt ligand, is ubiquitously misexpressed (Fig. S2G–P). Indeed, the expression domain of β-catenin and Top-gal reporter expression were greatly expanded to the most anterior end of the SE in *Wnt8A*-misexpressed embryos compared to the wild-type embryos (Fig. S2A″–C″,D‴–F‴), indicating enhancement of canonical Wnt activity. In accord with the hypothesis that the specification between NE and SE fates is altered, the NE markers Sox2 and N-cadherin were down-regulated (Fig. S2K, L), whereas the SE markers *Tfap2c*, E-cadherin, and TROMAI were enhanced by *Wnt8A* misexpression (Fig. S2H′, I′, M–P). Together these findings strongly suggest that canonical Wnt signaling is crucial to specification of the SE fate from uncommitted progenitors in the neural plate border.

### 3.4 The *Grhl3* Gene is Expressed in the Uncommitted Neural Folds Upon Neural Tube Closure

To identify the transcriptional factors that can mediate the fate specification of SE cells, we re-surveyed the expression profiles of the DNA microarrays (Figs. S4, S5). In particular, we focused on SE-specific transcriptional factors under the control of canonical Wnt signaling. In the microarray, 151 genes were more highly expressed in the SE than in the NE (fold change > 3 and p-value < 0.05). Of these 151 genes, 20 are considered transcriptional factors, based on GO slim ontology (Fig. S5). We chose to focus on the *Grainyhead-like2* (*Grhl2*) transcription factor (*Tcfcp2I3*), which was upregulated over sixfold more in SE than in NE (Fig. S5; red letters).

The *Grhl* family of CP2 transcription factors consists of important regulators of epidermal development in diverse species: *grainyhead* (*grh*) is a key determinant of epidermal barrier formation, wound healing, tube size, and neuroblast development in *Drosophila*, and *Grhl* genes play crucial roles in epidermal differentiation as well as neural tube closure in mammals (Pyrgaki et al., 2011, Rifat et al., 2010, Werth et al., 2010). In addition to *Grhl2*, *Grhl3* is also expressed during epidermal maturation (Auden et al., 2006) and genetically interacts with the *Grhl2* gene to achieve neural tube closure (Rifat et al., 2010). Hence, *Grhl3* is likely to be another candidate for SE specification via Wnt signaling.

**Fig. 5:**
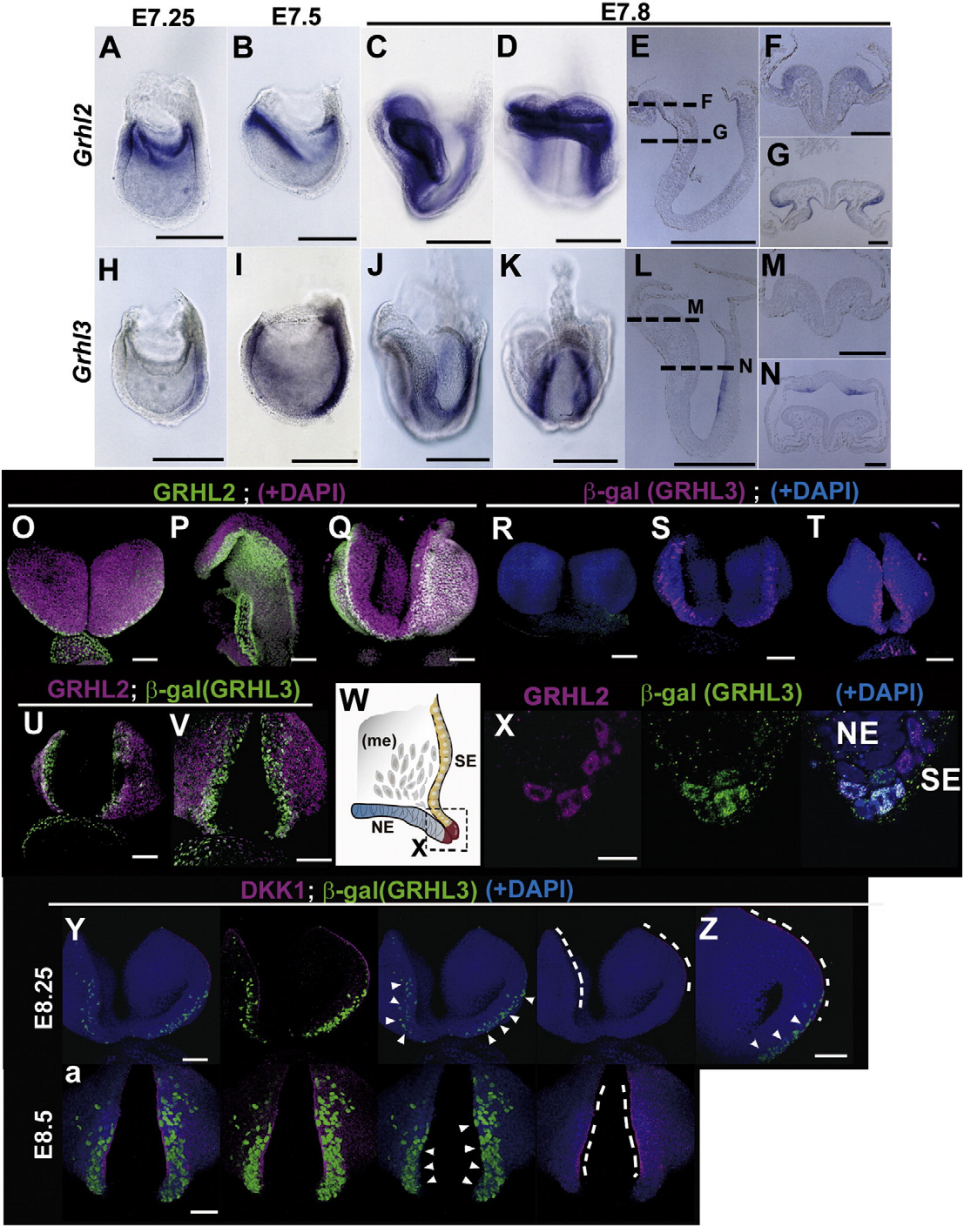
*Grhl* genes are expressed in the prospective SE. (A–N) *In situ* hybridization of whole-mount embryos (A–D, H–K), sagittal sections (E, L), and transverse sections (F, G, M, N) at E7.25 (A, H), E7.5 (B, I), and E7.8 (C–G, J–N). Lateral view (A–C, H–J), frontal view (D), and caudal view (K). (O-a) Whole-mount immunofluorescent staining (O–V, Y-a) and sectioning image (X) at E8.0 (O, P, R), E8.25 (S, U, Y), and E8.5 (Q, T, V, Z). (W) A section plane (X) is indicated in the schematic. Expression of GRHL2 (O–Q: GRHL2, green; nuclei, magenta) and β-gal antibody (R–T: GRHL3, magenta; nuclei, blue) in wild-type (O–Q) and *Grhl3^cre-nZ/+^* (R-a). (U–X) *β*-gal (GRHL3; green) and GRHL2 (magenta) merged with DAPI staining (nuclei, blue). (Y-a) DKK1 (magenta; dotted lines), β-gal (GRHL3, green; arrowheads) and nuclei (DAPI, blue). Scale bars: 300 μm in A–E, H–L; 100 μm in F, G, M–V, Y-a; 10 μm in X.

To test the possibility that these *Grhl* genes can contribute to the fate specification of SE cells in conjunction with neural tube closure, we first examined the expression of *Grhl2* and *Grhl3* transcripts with *in situ* hybridization from the late gastrula to neurula stages (Fig. 5A–N). At the early to late bud stages, *Grhl2* transcripts were detected in the most proximal-embryonic region, and the expression domain became restricted to the prospective border of the NE and SE in the rostral region (Fig. 5A–G), whereas *Grhl3* was preferentially expressed in the more caudal border between the NE and SE, where the levels of *Grhl2* transcripts were rather low (Fig. 5H–N).

Considering that canonical Wnt promotes the SE fate within the uncommitted neural plate border (Fig. 4, Fig. S2), we tested whether *Grhl2* and –*3* factors are upregulated in the neural plate border in conjunction with neural tube closure (Fig. 5O–a). *Grhl2* proteins were found broadly throughout the cranial SE cells already from the head fold stage (Fig. 5O–Q; green) before the neurulation stage and continuing after the neural tube closure stage, mostly co-localized to the TROMAI-positive cells. On the other hand, *Grhl3*-β-gal expression, which is marked by Cre-IRES-NLS *LacZ* driven by the *Grhl3* locus (Camerer et al., 2010), was not detected at the early somite stage (Fig. 5R). At the initiation of neural plate folding, *Grhl3*-β-gal appeared to form a line of dots along the border, and the expression appeared to increase in the midline region from the most rostral to the caudal direction (Fig. 5S, T; magenta). Compared to GRHL2, GRHL3 was evident in the proximity of the neural plate border between SE and NE (Fig. 5U–X).

These findings indicated that GRHL2 is expressed in SE cells from the early stage irrespective of the epithelial fusion of the neural folds and that GRHL3 is upregulated in the neural folds in conjunction with the neural tube closure event. To further address whether the DKK1-positive sharp boundary can demarcate the GRHL3-positive cells, we examined the expression of GRHL3 and DKK1 proteins and found that the *Grhl3* products were located consistently on the SE side of the sharp boundary delineated by DKK1 products (Fig. 5Y, Z, a). These expression data suggest the possibility that *Grhl3* mediates the SE fate specification of uncommitted progenitor cells via canonical Wnt signaling in coordination with the dorsal midline epithelial fusion of neural folds.

### 3.5 The *Grhl3* Gene is Necessary to Specify the SE Fate of Uncommitted Progenitors

**Fig. 6:**
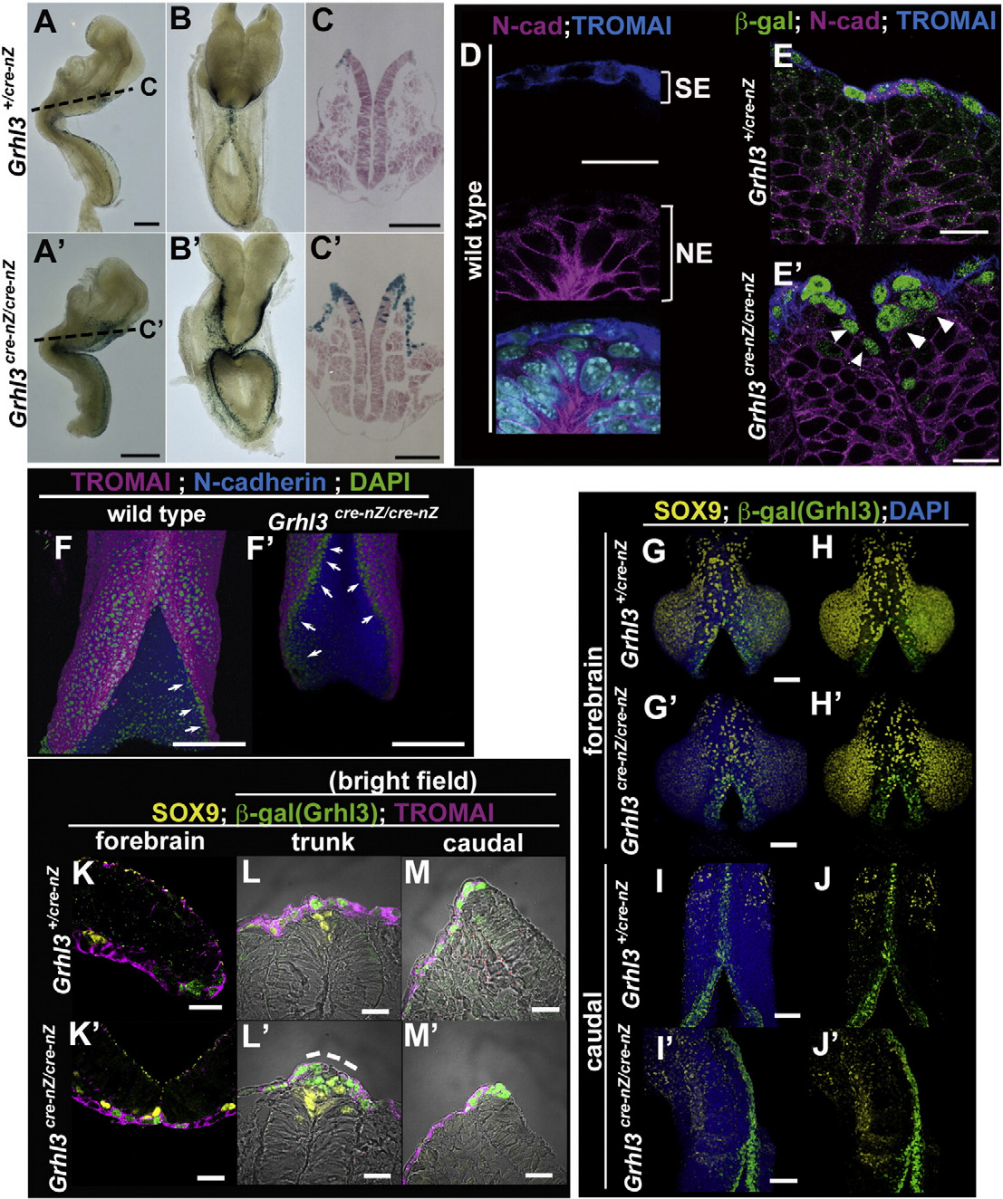
SE in the neural plate border is converted into the NE fate in the *Grhl3* mutant embryos. (A–C′) Lineage analysis of *Grhl3* expression in *Grhl3^+/cre-nZ^* (A–C) and *Grhl3 ^cre-nZ/cre-nZ^* (A′–C′) embryos at E8.25 with X-gal staining. Transverse sections counterstained with eosin (C, C′). (D, E, E′) Immunohistochemical analyses of whole embryo (D) and frozen section (E, E′) at E8.25 in wild-type (D), *Grhl3^cre-nZ/+^* (E), and *Grhl3^cre-nZ/cre-nZ^* (E′) embryos. β-gal (GRHL3, green), N-cadherin (magenta), TROMAI (blue), and DAPI (cyan). β-gal-positive cells express N-cadherin in addition to TROMAI within the SE layer in *Grhl3* heterozygous mutant embryos (E). β-gal-positive cells are mislocalized within the NE tissue in *Grhl3* null embryos (E′, white arrowheads). (F, F′) Immunohistochemical analyses of whole embryos at E8.5 in wild-type (F) *Grhl3^cre-nZ/cre-nZ^* (F′). DAPI (green), N-cadherin (blue), and TROMAI (magenta) in F and F′. N-cadherin-negative and TROMAI-negative cells correspond to uncommitted neural border progenitor cells (green; arrows in F and F′). (G–M′) Immunohistochemical analysis with a neural crest marker, SOX9 using whole-mount embryos (G–J′) and frozen sections (K–M′) in *Grhl3^cre-nZ/+^* (G–M) and *Grhl3^cre-nZ/cre-nZ^* (G′–M′). SOX9 (yellow), β-gal (GRHL3, green), TROMAI (magenta) and nuclei (DAPI, blue). Regarding cell behaviors of the SOX9-positive neural crest, a clear difference was not detected between heterozygous and homozygous mutant embryos. Scale bars: 300 μm in A, A′; 200 μm in D–F′; 100 μm in C, C′, G–J′; 20 μm in K–M′.

To address whether *Grhl3* promotes the SE fate of uncommitted progenitors marked by DKK1/KREMEN1, we examined the lineage of SE cells in the *Grhl3* null mutant background (Camerer et al., 2010). Notably, in the absence of *Grhl3* function (*Grhl3^cre-nZ/cre-nZ^*), the prospective SE cells, which are labeled by IRES β-gal expression driven by the endogenous *Grhl3* promoter, were ectopically found within the neural tissue (Fig. 6A′–C′). Next, to examine whether the mis-localized β-gal-positive cells possess NE or SE character, we performed a marker analysis with immunohistochemistry (Fig. 6D–E′). Normally, at E8.5 the neural tube at the levels of the hindbrain and anterior spinal cord is largely closed and TROMAI-positive SE cells and N-cadherin-positive NE cells are exclusively separated into surface and neural tube layers in wild-type embryos (Fig. 6D). However, β-gal-positive *Grhl3* heterozygous mutant cells within the SE region ectopically expressed N-cadherin in addition to TROMAI (Fig. 6E).

Moreover, β-gal-positive *Grhl3*-deficient cells were often observed within NE tissues in *Grhl3^cre-nZ/cre-nZ^* embryos and appeared to express N-cadherin but not TROMAI (Fig. 6E′). These results suggest that β-gal-positive cells appeared to specify into NE cells when the *Grhl3* gene dosage was reduced, and thus that *Grhl3*-deficient cells tend to be mis-specified into the NE fate. In addition, uncommitted progenitor cells, which are specified into neither NE nor SE and which are marked by TROMAI-negative and N-cadherin-negative cells, were increased in *Grhl3*-deficient embryos (Fig. 6F′).

To determine whether GRHL3-positive cells are relevant to neural crest cells, we investigated SOX9 expression (Fig. 6G–M′). Most of the GRHL3/β-gal-positive cells did not contribute Sox9-expressing neural crest cells in the *Grhl3* heterozygous or homozygous mutant embryos at the level of the forebrain and caudal spinal cord (Fig. 6G–M′). In addition, these neural crest cells appeared to be unaffected by *Grhl3*-deficiency (Fig. 6G–M′). Taken together, the present results indicate that *Grhl3* primarily participates in SE fate specification in the neural plate border in conjunction with the epithelial fusion of neural folds.

### 3.6 *Grhl3* Interacts Genetically with the Canonical Wnt Pathway in Terms of SE Specification

**Fig. 7:**
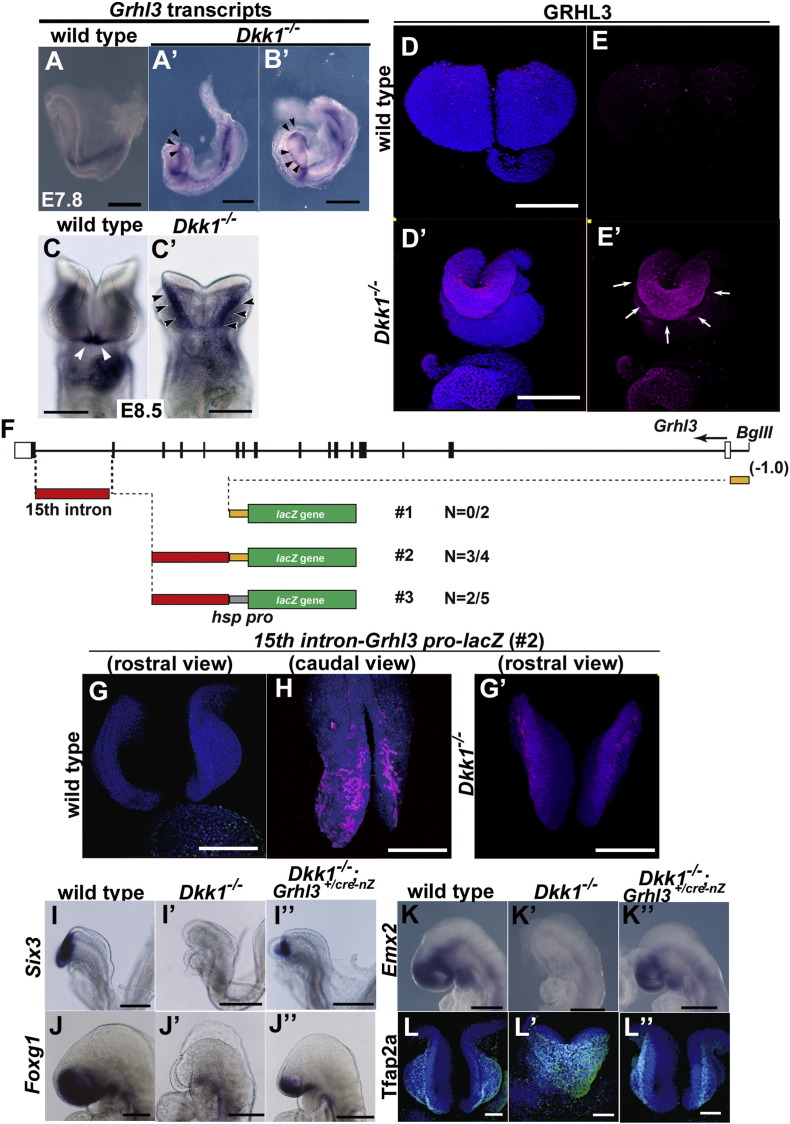
The expression of the *Grhl3* gene in the neural plate border is controlled by *Dkk1* during primary neurulation. (A–C′) *In situ* hybridization of whole-mount embryos at E7.8 (A–B′) and E8.5 (C, C′). Expression of *Grhl3* transcripts in wild-type (A, C) and *Dkk1*^−/−^ (A′–C′). (D, E′) Immunohistochemistry of whole-mount wild-type (D, E) and *Dkk1*^−/−^ embryos (D′, E′). GRHL3 (magenta) and nuclei (DAPI, blue). Both *Grhl3* transcripts and proteins are ectopically expressed in the rostral neural plate border (A′–E′). (F) Schematic diagram of the mouse *Grhl3*-endogeneous (from − 1.0 kb to 0)/or *hsp* promoter construct. The 15th intron region (2.35 kb; *Sal*I–*Bgl*II), which is conserved among vertebrates, fused with the β-gal cassette. (G, G′, H) The expression of β-gal proteins carrying the transgenic #2 construct appears to recapitulate the endogenous *Grhl3* expression in the wild-type (G, H) and *Dkk1*^−/−^ (G′) embryos. (I–L″) The reduction of *Grhl3* gene dosage can partly rescue the forebrain defects in *Dkk1*^−/−^ embryos at E8.25 (I–I″) and E8.5 (J–L″). Whole-mount *in situ* hybridization (I–K″) and immunohistochemistry (L–L″) in wild-type (I–L), *Dkk1*^−/−^ (I′–L′), and *Dkk1*^−/−^*;Grhl3*^+*/cre-nZ*^ (I″–L″). *Six3* (I–I″), *Foxg1* (J–J″), *Emx2* (K–K″), and TFAP2A (L–L″). Scale bars: 300 μm in A–C′, I–K″; 200 μm in D–E′, G, G′, H; 100 μm in L–L″.

To validate whether *Grhl3* expression is controlled by Wnt signaling, we first analyzed this expression in *Dkk1*-deficient embryos (Fig. 7A–E′). In the *Dkk1*^−/−^ embryos, the *Grhl3* transcripts and protein products were ectopically expanded to the entire neural plate border at the forebrain level (Fig. 7A–E′; arrowheads in A′–C′; arrows in E′). Next, to investigate whether the regulatory mechanism by Wnt signaling depends on the transcription level through *cis*-elements of the *Grhl3* gene, we surveyed the mouse *Grhl3* intron 15th, which is highly conserved among vertebrates and carries the enhancer activity in the zebrafish periderm cells at the early gastrula stage (de la Garza et al., 2013).

The transgene construct that harbors the conserved *Grhl3* 15th intron region linked to a reporter gene, *lacZ*, was constructed, after which transgenic mouse lines were generated and the β-gal reporter activity of F1 transgenic embryos was analyzed (Fig. 7F–G′). Consequently, the transgene displayed the enhancer activity in the SE at the levels of the hindbrain and spinal cord, but not at that of the forebrain, which appears to recapitulate the endogenous *Grhl3-lacZ* knocked-in expression patterns at E8.25 (Figs. 6A–C, 7G, H). To further test whether this enhancer activity can be affected by *Dkk1* expression, we examined the reporter activity in the *Dkk1*-deficient-background and found that it was enhanced in the SE of the forebrain as well as in those of the hindbrain and spinal cord (Fig. 7G′). These precise expression studies together suggest that *Grhl3* expression in the neural plate border during neural tube closure is regulated by canonical Wnt signaling at the level of transcription.

To further investigate whether phenotypic defects in the *Dkk1*-deficient embryos are partly brought about by the ectopic expression of the *Grhl3* gene due to increased canonical Wnt signaling, we crossed *Grhl3* mutant mice with *Dkk1* mutant mice and examined whether a reduction in the *Grhl3* gene dosage could rescue the anterior defects of *Dkk1*-deficient embryos (Fig. 7I–L″). Consequently, anterior NE marked by *Six3*, *Foxg1*, and *Emx2* was partly restored in *Dkk1*^−/−^ embryos by reducing one copy of the *Grhl3* gene (Fig. 7I″–K″). Concurrent with the alteration in NE markers, the expanded expression of *Tfap2a*, an SE marker at the level of the forebrain (Fig. 7L′), was reduced toward the normal condition in the *Dkk1*^−/−^; *Grhl3^+/cre-nZ^* embryos (Fig. 7L″). These findings indicate that the reduction in *Grhl3* activity can partly compensate for a failure in anterior development due to *Dkk1* deficiency. They also support the idea that *Dkk1* promotes NE specification by repressing *Grhl3* expression in the neural plate border.

**Fig. 8:**
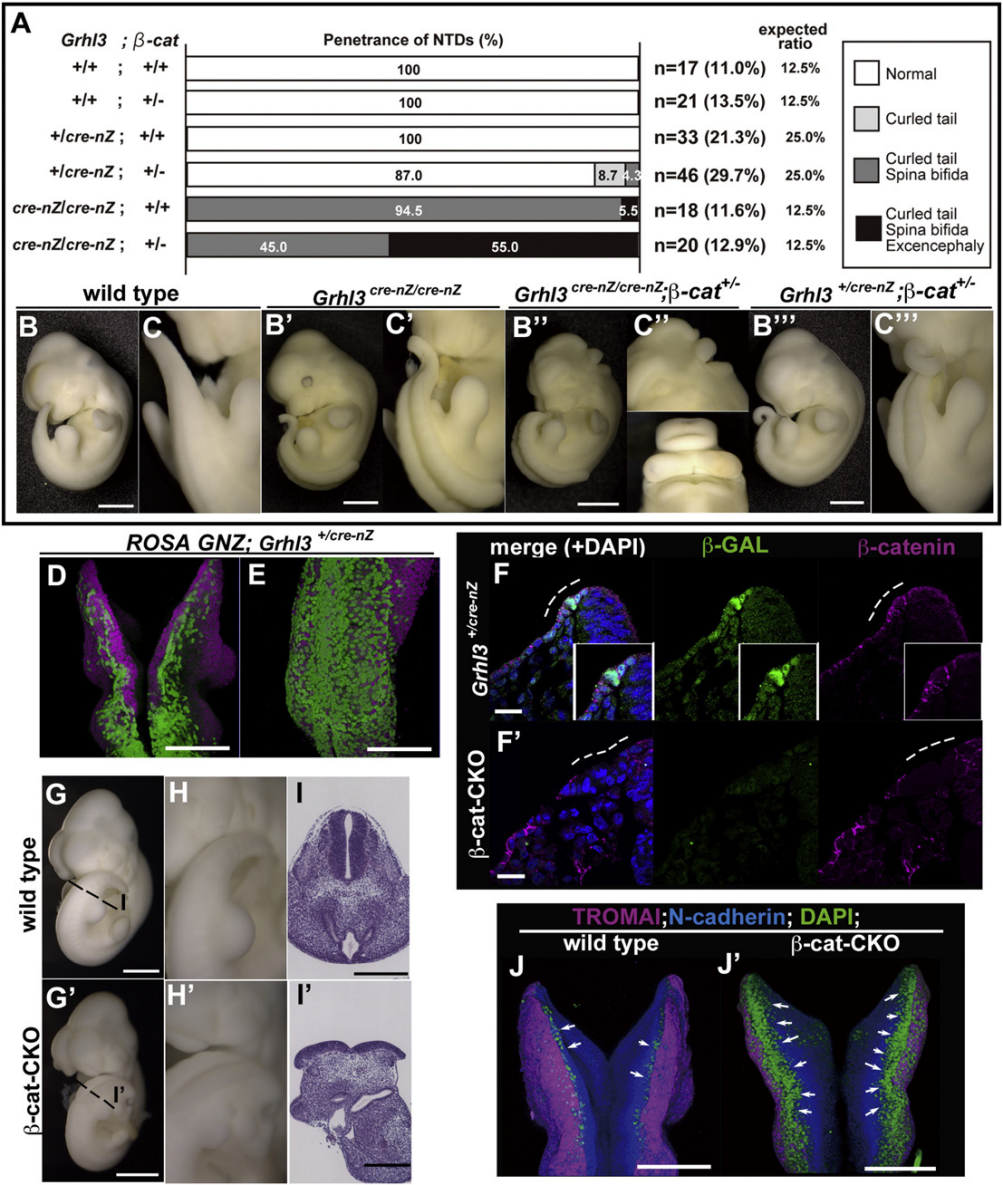
*Grhl3* genetically interacts with *β-catenin* for the SE specification. (A) The number of E11.5 embryos of each genotype from mating crosses between *Grhl3^+/cre-nZ^*; *β-catenin^+/−^* and *Grhl3^+/cre-nZ^* mice and the frequency with which penetrance shows neural tube defects, respectively (n = 155). (B–C‴) Neural tube defects of the compound mutants of *Grhl3*; *β-catenin*. Gross appearance of *Grhl3* mutant and *Grhl3*; *β-catenin* double-mutant embryos. Wild-type (B, C), *Grhl3^cre-nZ/cre-nZ^* (B′, C′), *Grhl3^cre-nZ/cre-nZ^*; *β-catenin*^*+/*−^ (B″, C″), and *Grhl3^+/cre-nZ^*; *β-catenin*^*+/*−^ (B‴, C‴). *Grhl3*; *β-catenin* double heterozygous mutant embryos exhibit spina bifida (B‴, C‴), whereas the *Grhl3* single heterozygous mutant has no defects by appearance (A). Moreover, the *Grhl3^cre-nZ/cre-nZ^*; *β-catenin^+/−^* embryos display exencephaly in addition to spina bifida and curled tail (A, B″, C″). (D, E) Detection of *Grhl3-cre* recombinase-active cells labeled by GFP protein expression (green) with whole-mount immunohistochemistry. *Grhl3-cre-nZ* mice, which were produced by the targeted insertion of the *Cre* gene into the *Grhl3* locus, are crossed with *ROSA-GNZ* mice (Stoller et al., 2008). Dorsal views of doubly transgenic offspring showing GFP expression only in the SE, not in the NE at the hindbrain (D) and trunk (E) levels. (F, F′) The production of *β-catenin* deletion in *Grhl3*-positive SE by crossing *β-catenin^flox/+^* or *β-catenin^flox/flox^* with *Grhl3-cre-nZ* mice. β-gal (GRHL3, green), β-catenin (magenta), and nuclei (DAPI, blue). The expression of *β-catenin* proteins is specifically lost in the *Grhl3*-*β*-*gal*-positive cells of the conditional knockout embryo (F, F′; dotted line). (G–I′) The gross morphology (G–H′) and histology (I, I′) in the wild-type (G–I) and conditional β-catenin knockout (G′–I′) embryos at E10.5. *β-catenin* conditional knockout embryos show spina bifida (G′–H′). (J, J′) Immunohistochemistry of *β-catenin* conditional knockout embryos. TROMAI (magenta), N-cadherin (blue), and nuclei (DAPI, green). Progenitor cells, which express neither NE nor SE markers (green), are expanded by *β-catenin* conditional mutation (J′; arrows). Scale bars: 1 mm in B–C‴; 500 μm in G, G′; 300 μm in I, I′; 200 μm in D, E, J, J′; 20 μm in F, F′.

To test the relationship between canonical Wnt signaling and *Grhl3* genetically in terms of neural tube closure, we generated the compound mutant *Grhl3*; *β-catenin* by crossing double heterozygous mice (Fig. 8A–C‴). The *Grhl3^+/cre-nZ^* embryos did not exhibit neural tube defects, whereas the *Grhl3^cre-nZ/cre-nZ^* embryos displayed neural tube closure defects; all embryos had thoraco-lumbo-sacral spina bifida and a curled tail (100%), but only a small fraction of embryos also exhibited exencephaly (5.5%) (Fig. 8A, B′, C′) (Rifat et al., 2010, Ting et al., 2003). The *Grhl3^cre-nZ/cre-nZ^*; *β-catenin*^*+/*−^ embryos exhibited fully penetrant spina bifida, similar to *Grhl3^cre-nZ/cre-nZ^* alone, but more than half of the embryos displayed exencephaly (55.0%) (Fig. 8A, B″, C″). In addition, *Grhl3^+/cre-nZ^*; *β-catenin*^*+/*−^ mutant embryos showed the thoraco-lumbo-sacral spina bifida phenotype, although penetrance was not high (curled tail 8.7%; curled tail and spina bifida 4.3%) (Fig. 8A, B‴, C‴). The enhanced manifestations of neural tube defects in the *Grhl3* and *β-catenin* double-mutant embryos provide compelling evidence of a positive genetic interaction between the canonical Wnt pathway and *Grhl3*. Our data further support the notion that canonical Wnt signaling is epistatic to the *Grhl3* gene.

To further confirm whether *β-catenin* itself plays crucial roles in neural tube closure specifically within the GRHL3-positive uncommitted progenitor cells, we analyzed the conditional knockout (CKO) mutation of *floxed-β-catenin* driven by Cre recombinase from the *Grhl3* locus (Fig. 8D–J′). Consistent with the notion that *Grhl3* is expressed only in the prospective SE of the neural plate border at E8.5 (Fig. 5, Fig. 8D–F), β-catenin expression was specifically lost in the SE but not the NE of the neural plate border (Fig. 8F′). *β-catenin*-CKO embryos displayed spina bifida closely resembling that of the *Grhl3 ^cre-nZ/cre-nZ^* embryos (Fig. 8B′, C′, G′–I′).

**Fig. 9:**
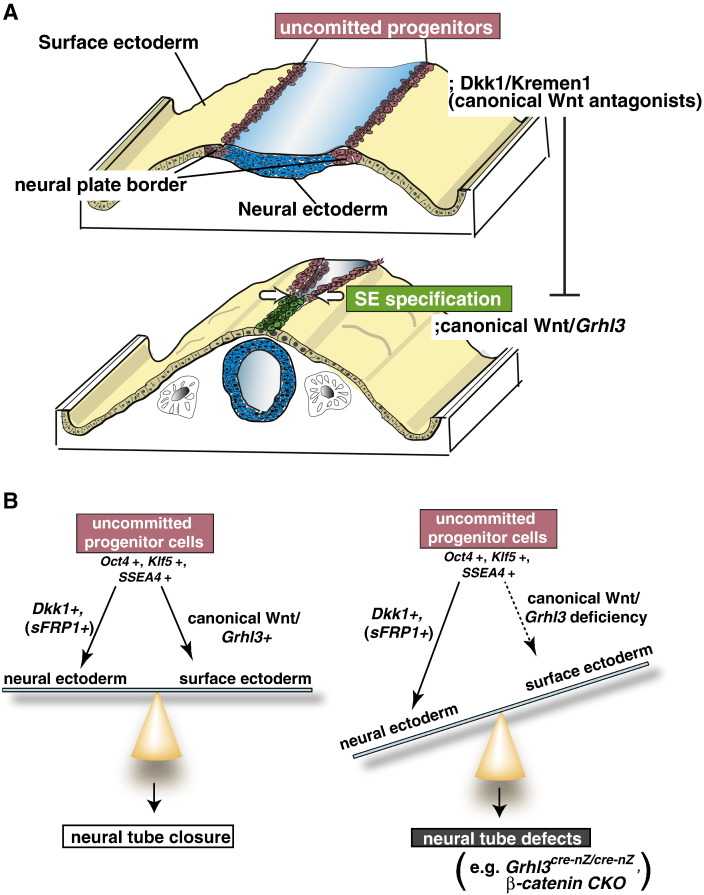
A schematic model of fate specification of uncommitted progenitors in the neural plate border/neural folds being coordinately coupled to neural tube closure. (A) A schematic model illustrating an intimate link between fate specification of the uncommitted progenitor cells and neural tube closure. Prior to the fusion of neural folds, the neural plate border cells between the NE (in blue) and SE (in yellow), which express DKK1/KREMEN1 (in red), are not specified into either the NE or SE fate as an uncommitted progenitor state marked by OCT4, KLF5 and SSEA4 (upper illustration). During the progression of neural tube closure, GRHL3-positive cells (in green) directed by canonical Wnt emerge coordinately in the vicinity of the neural plate border toward the identical direction of the neuropore closure, and then DKK1/KREMEN1-positive cells (in red) become restricted to the opposite side, where the neuropore is still open (lower illustration). (B) At the midline fusion of the neural folds, neural plate border cells lose the stem cell-like character and progressively specify into either SE or NE, regulated by canonical Wnt and its antagonists, respectively. In the *Grhl3^cre-nZ/cre-nZ^* and *β-catenin* conditional knockout embryos, uncommitted progenitor cells are expanded in the neural plate border and consequently they result in hyperplasia of NE with hypoplasia of SE, and consequently, the neural tube fails to close correctly.

In agreement with the above hypothesis that canonical Wnt signaling regulates the fate specification of uncommitted progenitors in the neural plate border, in the *β-catenin-*CKO embryos these progenitor cells, which are marked as TROMAI-negative and N-cadherin-negative cells, appeared to be increased compared to the wild type (Fig. 8J′). Together with the above two lines of evidence that *Grhl3* expression in the neural plate border is regulated at the transcriptional level by the canonical Wnt signaling (Fig. 7A–G′) and that the defects in fate specification observed in the *Dkk1*-deficient embryos can be restored by the removal of *Grhl3* gene dosage (Fig. 7I–L″), these findings strongly suggested that *Grhl3* and *β-catenin* genetically interact to achieve correct closure of the neural tube primarily in the fate specification of neural plate border cells. These results also support that the Wnt antagonists DKK1 and KREMEN1 may be required to maintain an uncommitted progenitor state and/or to keep away from SE specification for the neural plate border, whereas canonical Wnt participates in SE specification by activating *Grhl3* expression (Fig. 9, *see* Discussion).

## 4 Discussion

In this study, we observed genetic mechanisms underlying the fate specification of uncommitted progenitor cells in the neural plate border/neural fold coordinately coupled to neural tube closure in both time and space (Fig. 9). Notably, the *Grhl3* transcription factor can promote the SE fate as one of the downstream effectors of canonical Wnt signaling. Given that *Grhl3* directly contributes to neural tube closure genetically interacting with *β-catenin*, we propose that the fate specification of uncommitted progenitor cells in the neural plate border by canonical Wnt signaling and *Grhl3* is crucial for neural tube closure (Fig. 9).

The results of the present study showed that the specification of progenitor cells into the NE or SE fate is progressively controlled in both time and space by canonical Wnt and its antagonists during neural tube closure (Fig. 9A). Prior to the fusion of neural folds, the neural plate border possesses an uncommitted progenitor character such as Oct4, Klf5 and SSEA4 expression, which is specified into neither NE nor SE, and loses this undifferentiated character at the time of fusion (Fig. 1). Wnt antagonists DKK1/KREMEN1 contribute to the maintenance of the uncommitted progenitor character and/or to the avoidance of the SE specification by canonical Wnt during neurulation (Fig. 1, Fig. 3, Fig. 4). The properly balanced cell fate specification between NE and SE cells within the neural plate border during neurulation might lead to a progressive midline fusion of the neural folds and coincidental separation of the neural tube and SE, whereas a failure in cell fate specification in the neural plate border/neural fold might lead to neural tube defects (Fig. 9B).

*β-Catenin-*CKO mutation within the neural plate border, where the SE fate specification is altered, results in neural tube defects (Fig. 8G′–J′). Additionally, the gain and loss of *Dkk1* expression appear to affect the timing of neural tube closure (Fig. S2; unpublished data; C.K.-Y. and I.M.). Thus, we propose that the precise progressive mechanisms of fate specification in uncommitted progenitors involving canonical Wnt and its antagonists may determine the correct neural tube closure via epithelial development in the vertebrate embryo (Fig. 9).

Considering that cranial neural crest cells are delaminating from the non-neural ectoderm in the vicinity of the neural plate border/neural fold prior to neural tube closure in the mouse (Nichols, 1987, Weston and Thiery, 2015), it can be questioned whether the neural crest is relevant to uncommitted progenitor cells in the neural plate border and whether its population is primarily affected in our studies. Our study also supports the previous finding that a very few cranial neural crest cells appear to delaminate from uncommitted progenitors within the neural plate border at the forebrain level and from a considerable amount of neural crest cells at the hindbrain level (Fig. 2F, G). However, given that SOX9-negative uncommitted progenitor cells are still present at both the forebrain and hindbrain levels and that trunk neural crest cells emigrate from the dorsal neural tube after the completion of the fate specification of uncommitted progenitors (Fig. 2E–I), the decision of fate specification into neural crest cells appears to be not directly linked to neural tube closure. Additionally, *Grhl3*-positive cells apparently contribute to few neural crest cells in the *Grhl3* heterozygous and homozygous mutant embryos (Fig. 6G–M′).

Concurrent with these cell behaviors, the formation of neural crest cells appeared not to be affected by *Dkk1*-misexpression or by *Dkk1* and *Grhl3* deficiency (Fig. 6G′–M′, Figs. S6, S7). Hence, although the fate decisions of neural crest cells appear to be controlled by stage-dependent differential responsiveness to canonical Wnt signaling activation after emigration in the mouse embryo (Hari et al., 2012), it is likely that the altered fate specification through canonical Wnt observed in this study is not due primarily to alterations of the fate decision of the neural crest.

*Grhl3* has been shown to play crucial roles in neural tube closure (Gustavsson et al., 2008, Rifat et al., 2010). However, there is little understanding of how *Grhl3* expression is controlled during primary neurulation. In the present study, *Grhl3* was shown to be one of the pivotal downstream effectors of canonical Wnt signaling for SE specification in uncommitted progenitors of the neural plate border/neural fold (Fig. 6, Fig. 7, Fig. 8, Fig. 9). This is in good agreement with the notion that *Grainyhead* transcription factors play conserved roles in epidermal differentiation in the animal kingdom (Wang and Samakovlis, 2012). Disruption of *Xenopus Grhl1* leads to defects in epidermal differentiation, as evidenced by the loss of expression of the keratin gene (Tao et al., 2005). Similarly, *Xenopus Grhl3* activates specific markers of superficial epidermal cells in deep proliferating progenitor cells (Chalmers et al., 2006). Mouse *Grhl1-*deficient embryos display defects in skin and hair, partly due to the reduction of desmosomal cadherins (Wilanowski et al., 2008). Consistent with that finding, in the present study the reduction of *Grhl3* activity appeared to inhibit SE specification (Fig. 6, Fig. 7, Fig. 8); i.e., neural plate border cells marked by *Grhl3*-expression tend to transform into NE cells in *Grhl3*-mutant embryos. These convergent lines of evidence support the idea that *Grhl3*-positive cells will differentiate into an SE fate by trans-activating SE-specific genes in a cell autonomous fashion and, conversely, that *Grhl3*-negative cells will tend to become NE cells in the neural plate border.

*Grhl3* plays crucial roles in the specification of SE fate of the uncommitted neural plate border for neural tube closure. The epithelial fusion and remodeling allow the reorganization of two multicellular structures spanning an anatomical and physiological gap to form a new, unified structure. The process of epithelial fusion involves cellular protrusions that extend from the leading edges of apposing SE cells (Waterman, 1976, Geelen and Langman, 1979, Pyrgaki et al., 2010). Indeed, fine structures of SE cells of the neural plate border appeared to be affected in the *Dkk1*-misexpressing embryos (Fig. S3). Given that apposing epithelial contact seems to be specific within neural folds, these cellular behaviors may be initiated by *Grhl3* as a crucial downstream effector of canonical Wnt signaling.

Considering our hypothesis that the fate specification of uncommitted progenitors is crucial for neural tube closure, it can be argued that *Grhl3*-deficient embryos display neural tube defects solely at the level of the caudal spinal cord and not at the forebrain and trunk levels (Rifat et al., 2010, Ting et al., 2003). We assume that the roles of *Grhl3* in fate specification might be complemented by *Grhl2* expression redundantly at the forebrain level (Fig. S8). To explore this possibility, we examined *Grhl2* expression in the *Grhl3* mutant background and found that at the forebrain level, *Grhl2* expression extended locally to the most medial SE (i.e., the neural plate border/neural fold) in the *Grhl3*-deficient embryos (Fig. S8A–D, E–H′), whereas at the caudal level the compensatory *Grhl2* expression in the neural plate border/neural fold is not evident (Fig. S8A–H).

Consistent with our hypothesis, the *Grhl3*-deficient embryos displayed cranial neural tube defects by the removal of one copy of the *Grhl2* gene dosage (Rifat et al., 2010). In addition, our misexpression studies with *Grhl2* revealed that *Grhl2* was able to induce the SE fate ectopically in the NE region (Fig. S8H′–P′), confirming the compensatory function of *Grhl2* in place of *Grhl3* for fate specification during neurulation. Consequently, we consider that the absence of both *Grhl2* and *Grhl3* expression in the neural plate border will cause neural tube defects.

Thus, together with the expression of stem cell markers and Wnt antagonists in the neural plate border (Fig. 1, Fig. 3), these findings lead us to propose that the properly balanced fate specification of uncommitted progenitor cells in the neural plate border/neural fold by canonical Wnt and one of its downstream effectors, *Grhl3*, may be crucial for correct neural tube closure (Fig. 9).

## Funding

This work was supported in part by a grant-in-aid for Scientific Research on Priority Areas and on Innovative Areas and Scientific Research (B) and (C) from the Ministry of Education, Culture, Sports, Science, and Technology, Japan, KAKENHI Grant Numbers, 24116713 26291053, 25461720; and by the Naito Foundation, the Mitsubishi Foundation, the Uehara Foundation and the Takeda Science Foundation. The funders had no role in the study design, data collection and analysis, decision to publish, or preparation of the manuscript.

## Authors' Contributions

CK-Y and IM initiated the research and planned most of the experiments. KE and CN generated the mouse *Dkk1*^flox/flox^ strain. CK-Y and KM performed most of the experiments. CK-Y, CN, and IM contributed to the writing of the manuscript.

## Conflict of Interest Disclosure

The authors have declared that they have no conflict of interest.
