# Supplementary material for: Fate Specification of Neural Plate Border by Canonical Wnt Signaling and Grhl3 is Crucial for Neural Tube Closure
Source: eBioMedicine. 2015 Apr 18;2(6):513–27. doi: 10.1016/j.ebiom.2015.04.012 (PMC4535158; doi:10.1016/j.ebiom.2015.04.012)
Supplement: Supplementary file 1 — Supplementary material. [file mmc1.docx]

**EBioMedicine**

**Appendix A. Supplementary data**

**Fate specification of neural plate border by canonical Wnt signaling and *Grhl3* is crucial for neural tube closure**

**Chiharu Kimura-Yoshida, Kyoko Mochida, Kristina Ellwanger, Christof Niehrs, and Isao Matsuo**

**Inventory of Supplemental Material:**

**Supplemental Figures**

**Figure S1** related to Figure 3

**Figure S2** related to Figure 4

**Figure S3** related to Figure 4

**Figure S4** related to Figure 5

**Figure S5** related to Figure 5

**Figure S6** related to Figures 2,4

**Figure S7** related to Figures 2,4

**Figure S8** related to Figures 6,8

**Figure S9** related to Figures 1-8

**
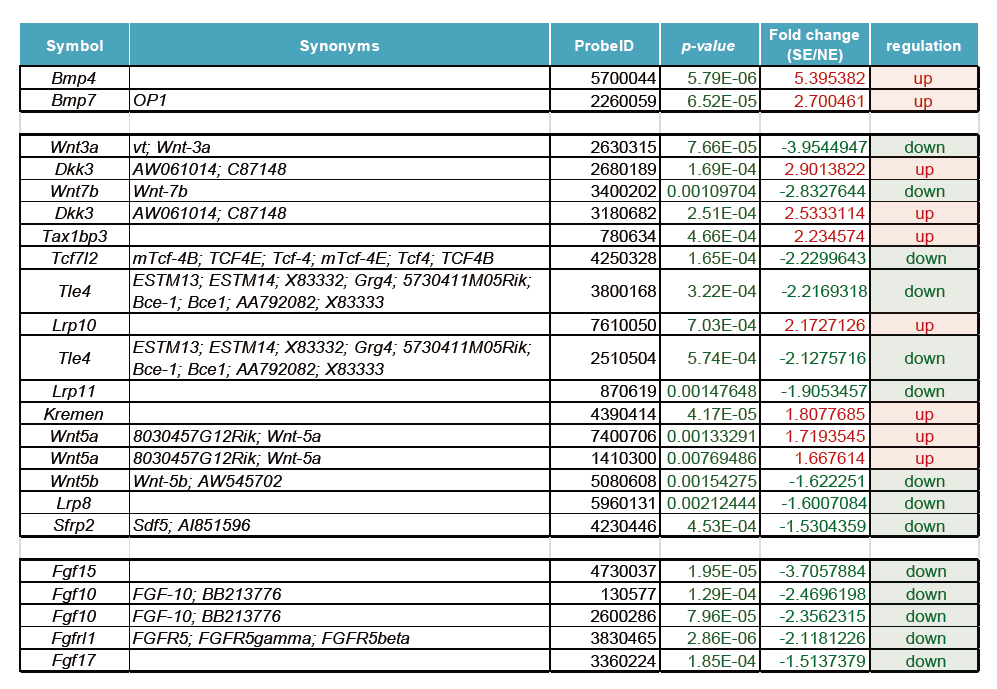
**

**Fig. S1. (related to Fig. 3) A list of growth-factor-related genes, BMP, Wnt and FGF identified by DNA microarray between surface ectoderm (SE) and neural ectoderm (NE) during neurulation.** The signaling-related molecules showed increased or decreased expression in the SE compared to the NE in E8.5 wild-type embryos. A fold change (−) indicates decreased expression in the SE.

**
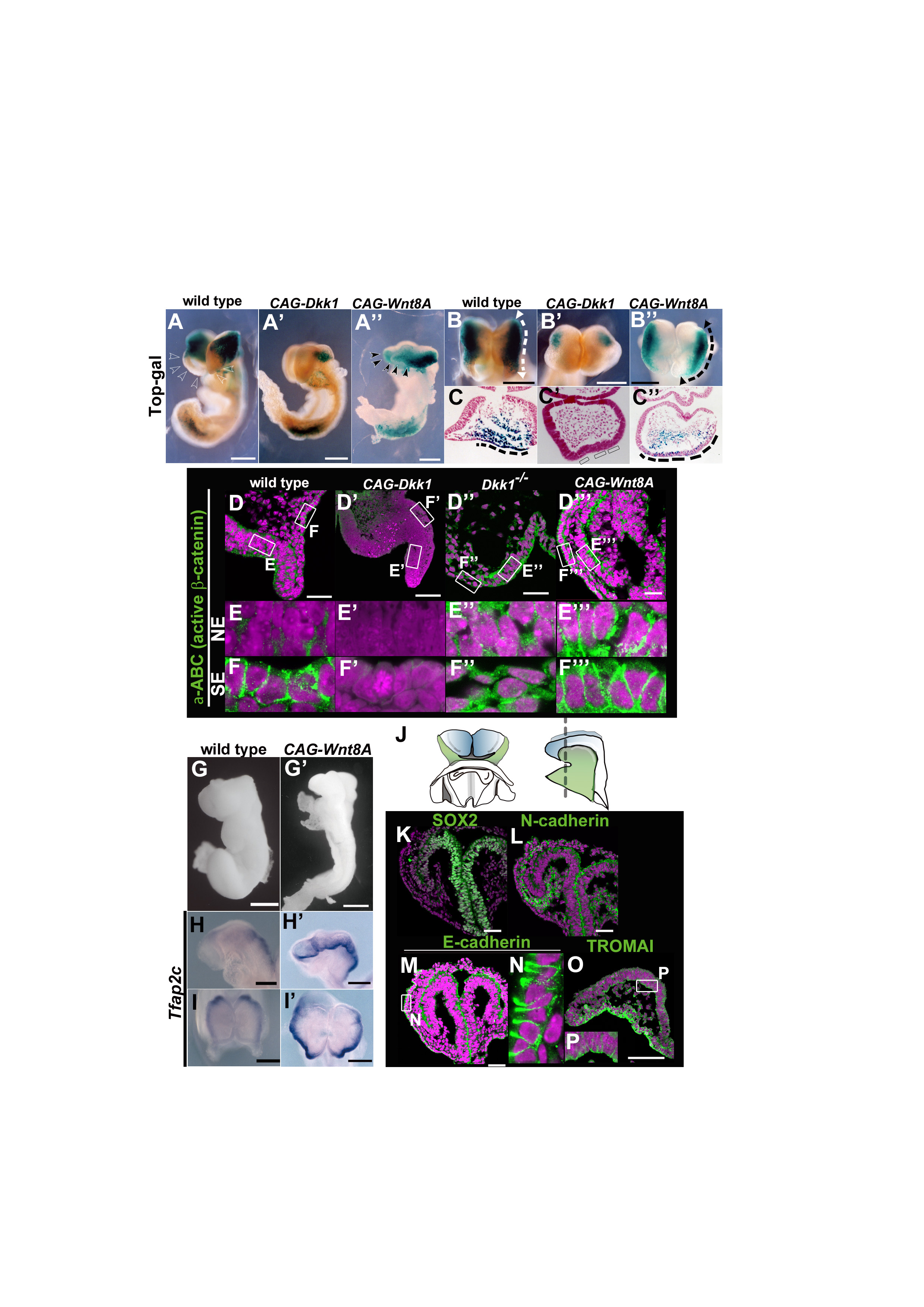
**

**Fig. S2. (related to Fig. 4) Analyses of canonical Wnt activity and molecular markers expression in transgenic and knockout embryos.** (A–Cʹʹ) Expression patterns of the Top-gal reporter in the wild-type (A–C), *CAG-Dkk1* (Aʹ–Cʹ), and *CAG-Wnt8A* embryos (Aʹʹ–Cʹʹ) at E8.5 with X-gal staining. Lateral views (A–Aʹʹ), dorsal views (B–Bʹʹ), and transverse sections (C–Cʹʹ). Top-gal expression is extended to the most anterior SE in the *CAG-Wnt8A* expression but not in the wild type (arrowheads and dotted lines in A,Aʹʹ, C,Cʹʹ). (D–Fʹʹʹ) Active form of β-catenin expression. Active β-catenin expression appears to be more intense in the SE than in the NE of the wild-type embryos (D–F). Misexpression of *Dkk1* exhibits reduced β-catenin activity (Dʹ–Fʹ), whereas *Dkk1* deficiency displays enhanced β-catenin activity in the anterior ectoderm (Dʹʹ–Fʹʹ). In *CAG-Wnt8A* embryos, active β-catenin is upregulated in the NE as well as in the SE (Dʹʹʹ–Fʹʹʹ). (G,Gʹ) Morphological features of wild type (G) and *CAG-Wnt8A* (Gʹ) at E8.5. (H–Iʹ) Whole-mount *in situ* hybridization of wild type (H,I) and *CAG-Wnt8A* (Hʹ,Iʹ) embryos with *Tfap2c,* an SE marker at E8.25. Lateral views (H,Hʹ) and dorsal views (I,Iʹ). (J) Schematics of planes of frontal sections in *CAG-Wnt8A* embryos. (K–P) Immunohistochemistry using SOX2 (K), N-cadherin (L), E-cadherin (M,N), and TROMAI (O,P) in *CAG-Wnt8A*. Scale bars: 300 μm in A–Bʹʹ,G–Iʹ; 100 μm in K–P; 50 μm in D–Dʹʹʹ.

**
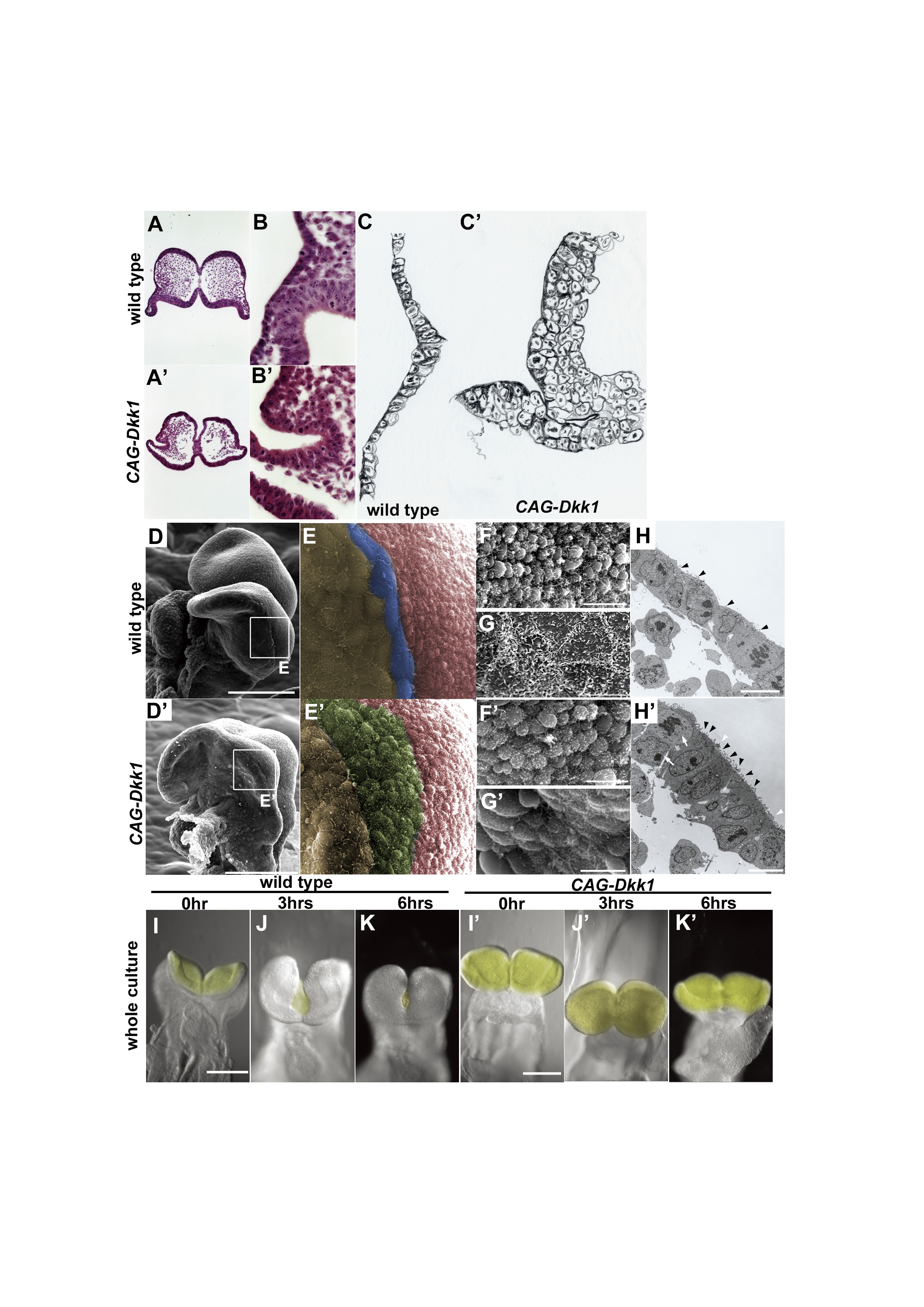
**

**Fig. S3. (related to Fig. 4) Morphological analyses of *CAG-Dkk1* embryos.** (A–Cʹ) Morphological features of wild-type (A–C) and *CAG-Dkk1* (Aʹ–Cʹ) embryos in hematoxylin-and-eosin-stained transverse sections at E8.5 (A–Bʹ). Multiple layers of the presumptive SE region in *CAG-Dkk1* embryos are formed (Bʹ, Cʹ), whereas there is only a simple layer of SE in wild-type embryos (B,C). (D–Hʹ) Fine structures of rostral NE and adjacent SE are shown with scanning electron microscopy (SEM) (D–Gʹ) and transmission electron microscopy (TEM) (H,Hʹ) of wild-type (D–H) and *CAG-Dkk1* embryos (Dʹ–Hʹ) at E8.5. NE (F,Fʹ) and SE (G,Gʹ). The neural plate border, “flattened cells (blue)” between the SE (yellow) and NE (red) is clearly observed in the wild-type embryos at E8.5 (E), whereas the border (Eʹ, green) between the SE and NE regions and bearing characteristics of NE-like cells is evident in the *CAG-Dkk1* embryo (Gʹ). Microvilli (arrowheads) and bleb-like protrusions (open arrowheads) are more abundant in the SE region of the *CAG-Dkk1* embryos than in that of the wild-type embryos (H,Hʹ). (I–Kʹ) External views of whole embryos after *in vitro* cultures (for 0, 3, and 6 h; 4–5 somites at the start of culture) are presented. Wild-type embryos appear to close the telencephalic neuropore mostly after the 6-h culture (K), whereas *CAG-Dkk1* embryos remain open (Kʹ) (opened neural plate in yellow). Scale bars: 200 μm in D,Dʹ, 100 μm in I,Iʹ; 10 μm in F–Hʹ.

**
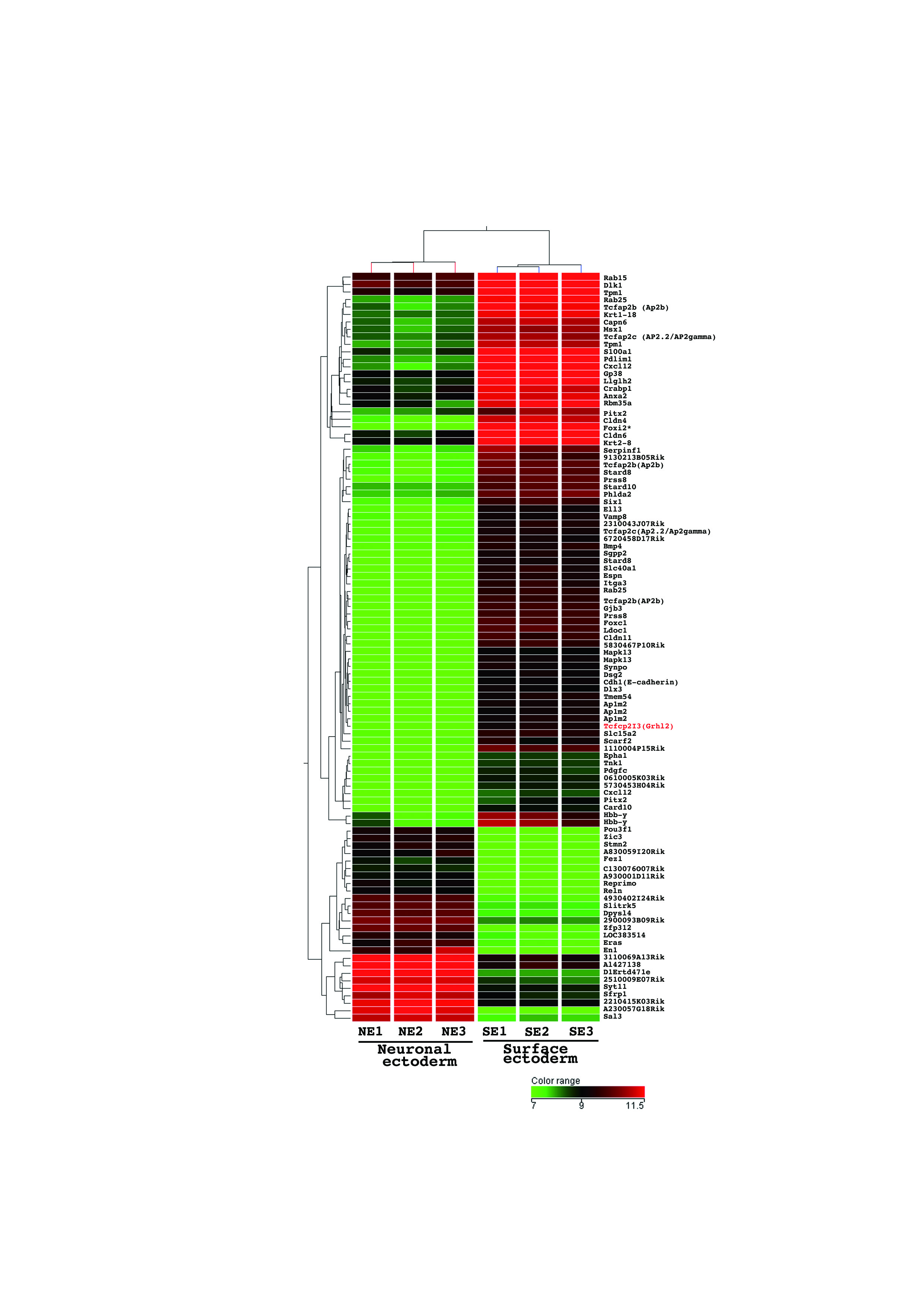
**

**Fig. S4. (related to Fig. 5) A screenshot of the microarray between surface ectoderm and neural ectoderm at E8.5.** Heatmap representation of the down- and up-regulated genes between the NE and SE (p<0.05). Red represents higher copy number and green represents lower copy number.

**
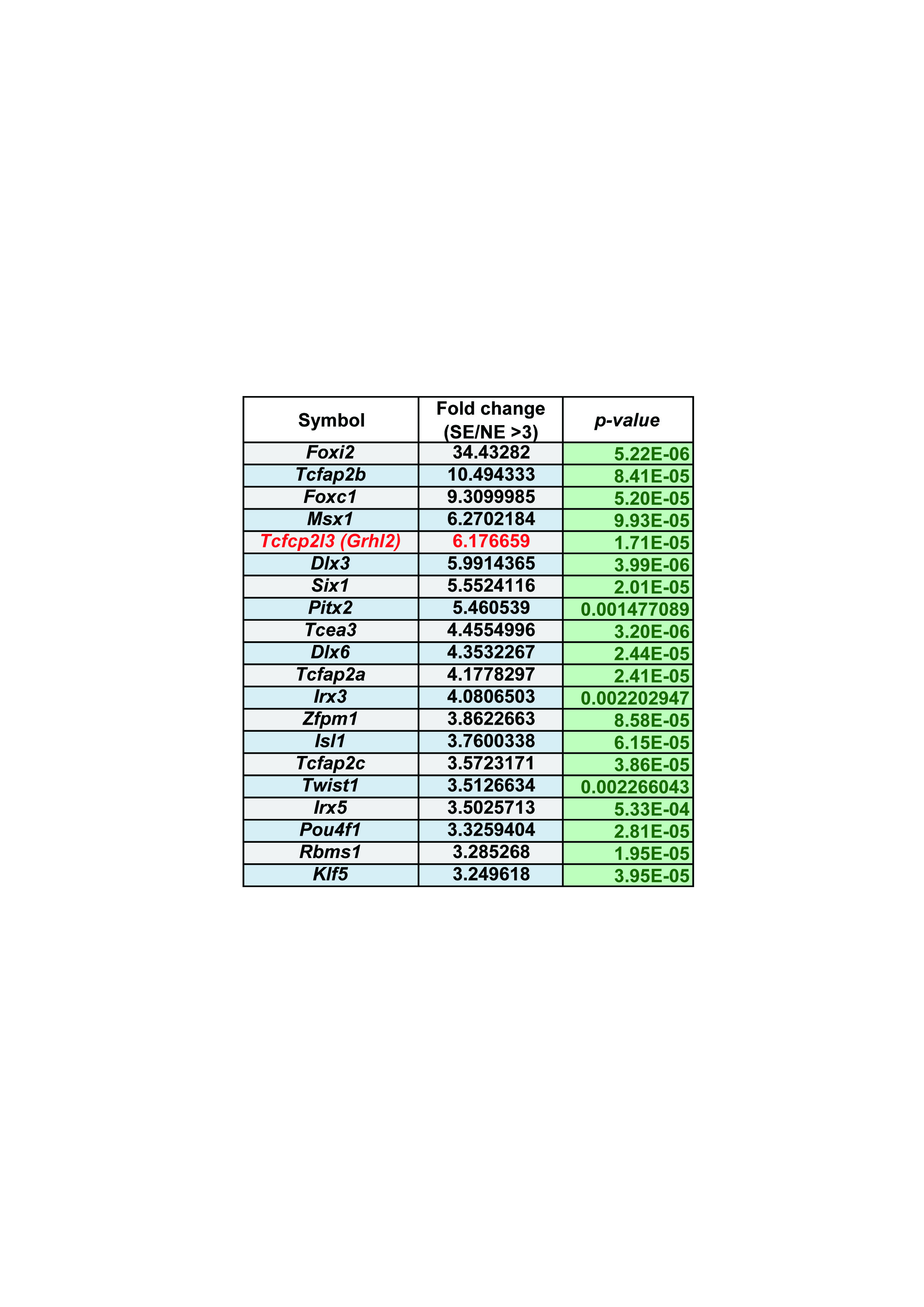
**

**Fig. S5. (related to Fig. 5) A list of transcription factors identified by DNA microarray in the SE compared to the NE.** Listed genes display > 3.0-fold enrichment in surface ectoderm vs. neural ectoderm cells (p<0.05). The most upregulated transcription factor is *Foxi2*.

**
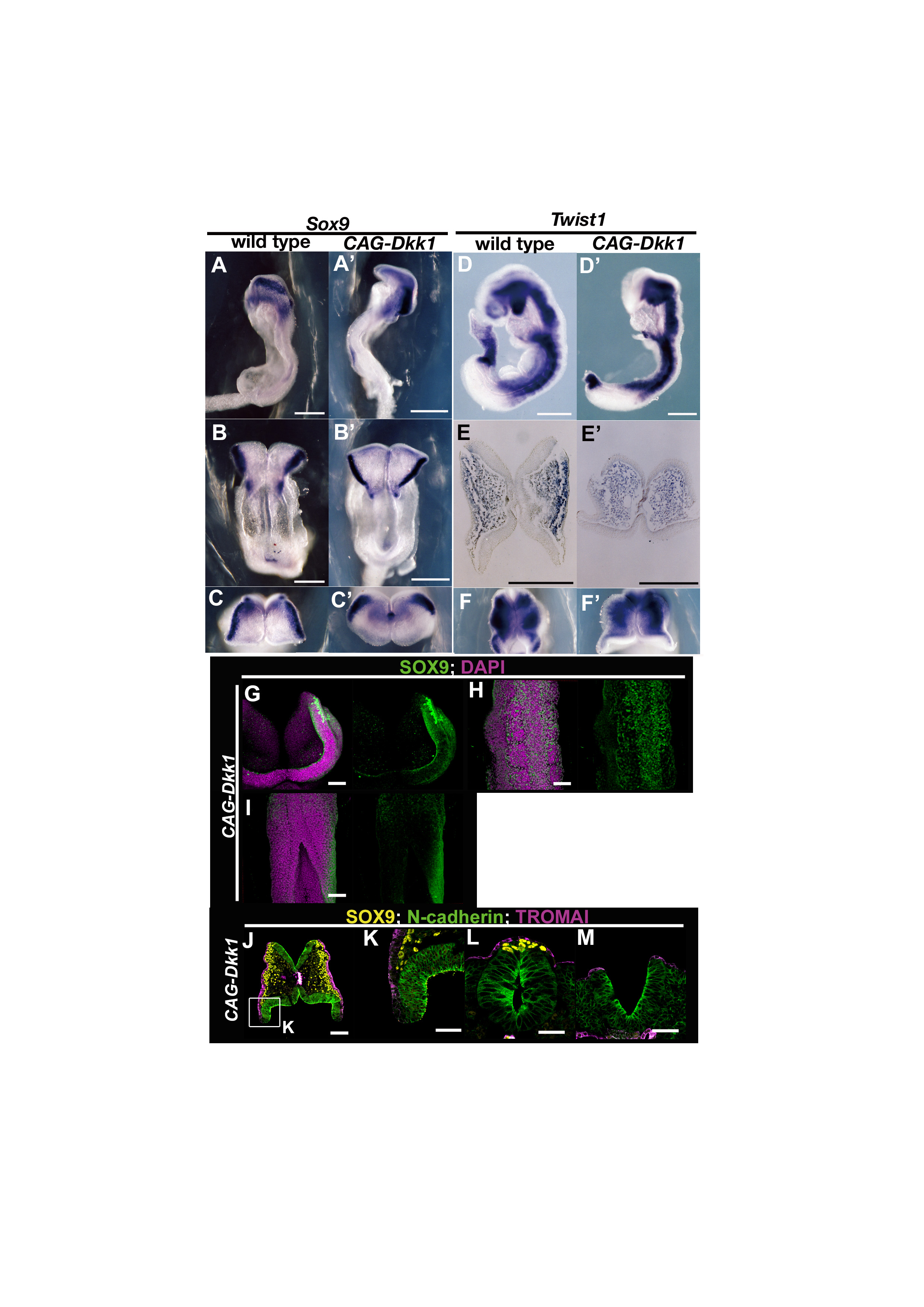
**

**Fig. S6. (related to Figs. 2, 4) The development of neural crest cells appeared to be unaffected in *CAG-Dkk1* embryos.** (A–Fʹ) Whole-mount *in situ* hybridization (A–Cʹ,D,Dʹ,F,Fʹ) and its transverse sections (E,Eʹ). Wild-type (A–F) and *CAG-Dkk1* (Aʹ–Fʹ) embryos at E8.5. *Sox9* (A–Cʹ) and *Twist1* (D–Fʹ). Lateral views (A,Aʹ, D,Dʹ) and dorsal views (B–Cʹ, F,Fʹ). (G–M) Whole-mount embryos (G–I) and frozen sections (J–M) with immunohistochemistry. SOX9 (green in G–I, yellow in J–M), N-cadherin (green in J–M), TROMAI (magenta in J–M) and DAPI (magenta in G–I) at E8.5. The expressions of *Sox9* and *Twist1*, which are markers of neural crest cells and cephalic mesenchyme, appeared to be unaltered in the *CAG-Dkk1* embryos (Aʹ–Fʹ, G–M). Scale bars: 300 μm in A–Bʹ, D–Eʹ; 100 μm in G–J; 50 μm in K–M.

**
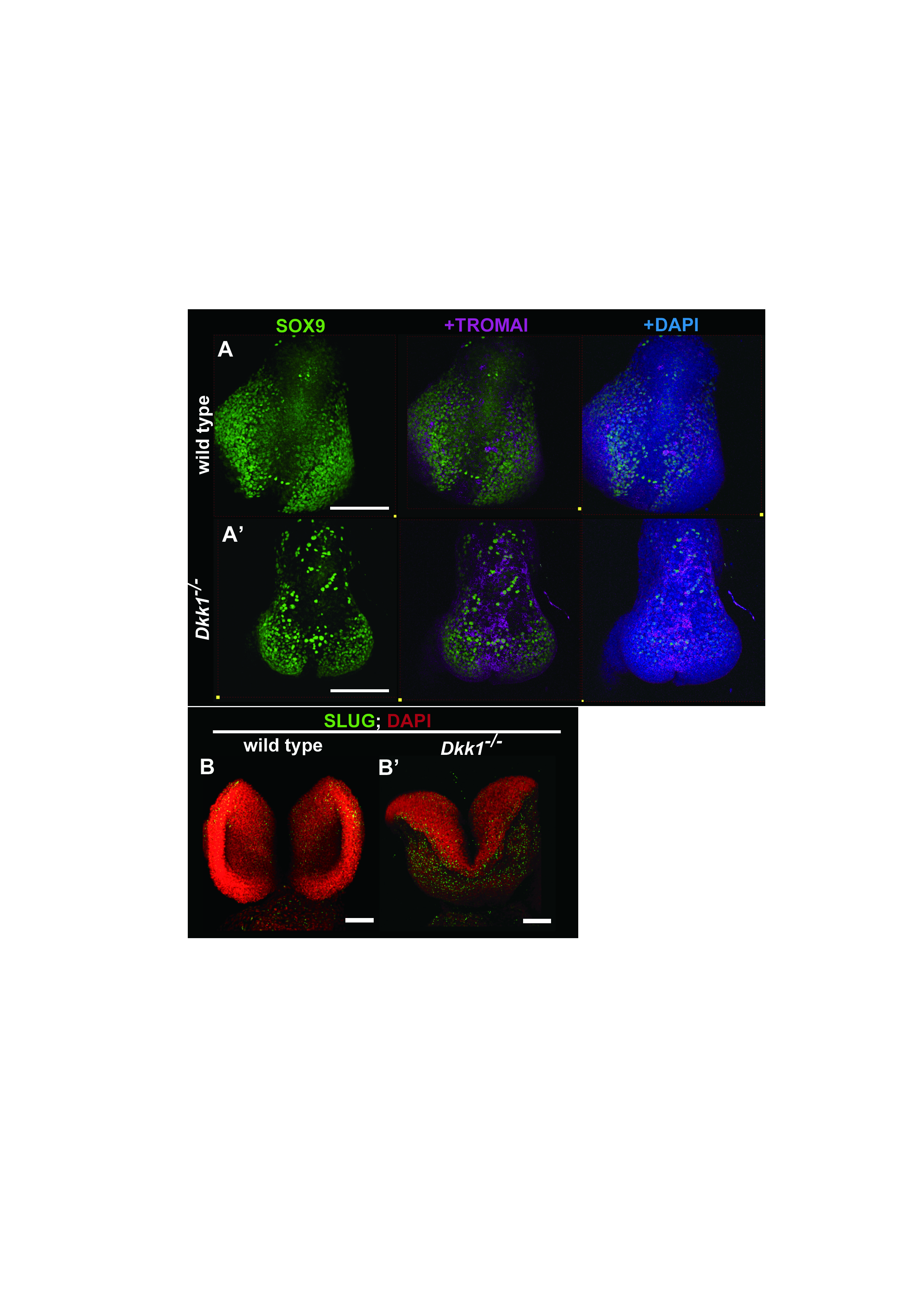
**

**Fig. S7. (related to Figs. 2, 4) Expression of neural crest markers in *Dkk1*^−/−^ embryos.** (A,Aʹ) Whole-mount immunohistochemistry with SOX9 (green in A,Aʹ), SLUG (green in B,Bʹ), TROMAI (magenta in A,Aʹ), and DAPI (nuclei; blue in A,Aʹ and red in B,Bʹ) in wild-type (A,B) and *Dkk1*^−/−^ embryos (Aʹ,Bʹ) at E8.5 (A–Bʹ). The SOX9- and SLUG-positive cells are normally localized in the *Dkk1*^−/−^ embryos, whereas TROMAI-positive SE cells are ectopically increased (Aʹ). Scale bars: 200 μm in A,Aʹ; 100 μm in B,Bʹ.

**
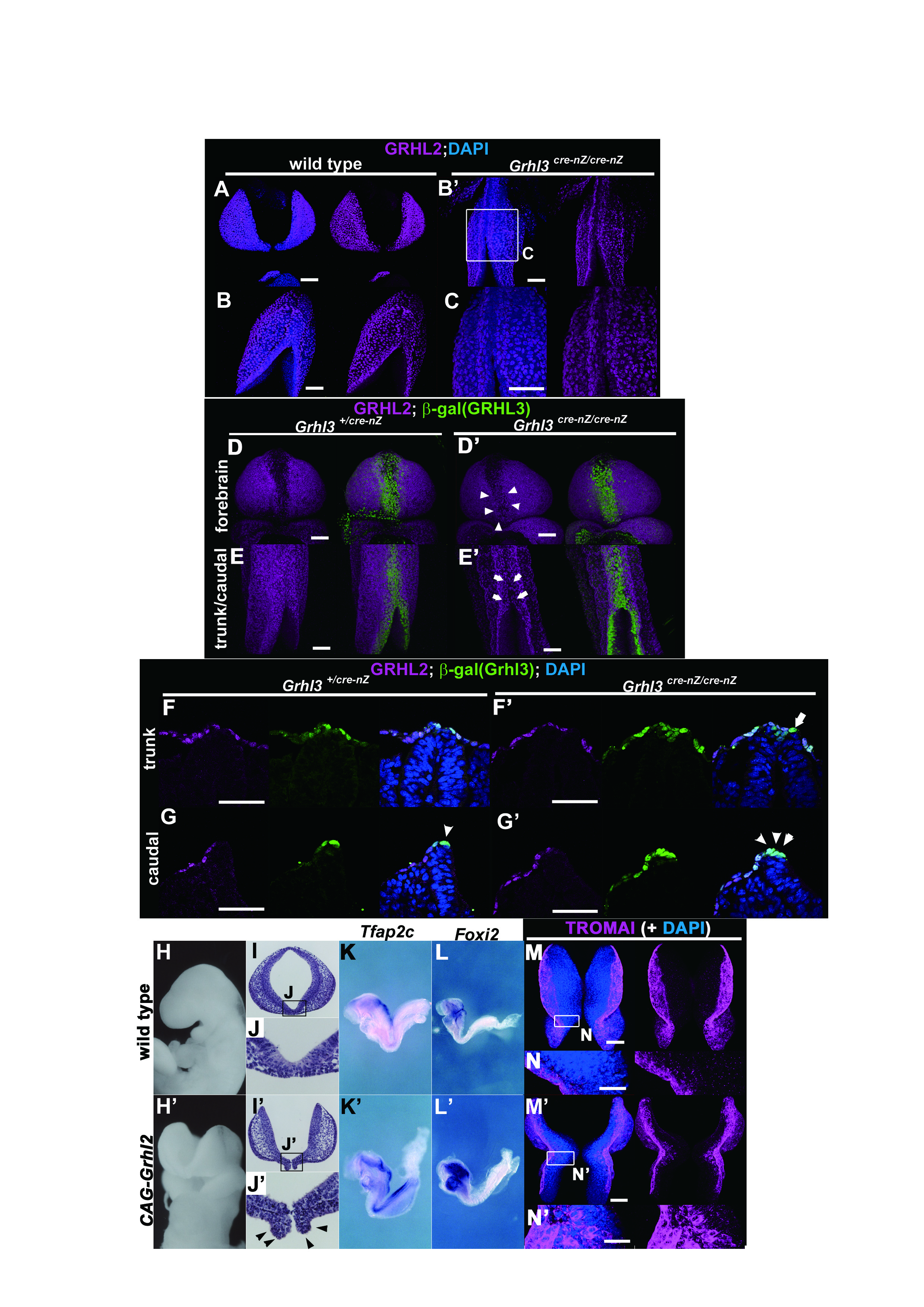
**

**Fig. S8. (related to Figs. 6, 8) Expressions of GRHL2 proteins during neurulation in the *Grhl3* mutant background and analyses with histology and molecular markers in *CAG-Grhl2* transgenic embryos.** (A–Gʹ) Whole-mount embryos and frozen sections with immunohistochemistry of GRHL2 (magenta in A–Gʹ), β-gal (D–Gʹ) and DAPI (blue in A–C, F–Gʹ) in wild-type (A,B), *Grhl3^+/cre-nZ^* (D–G) and *Grhl3^cre-nZ/cre-nZ^* (Dʹ–Gʹ) embryos at E8.5. At the forebrain to caudal levels, GRHL2 proteins are not expressed in the vicinity of the midline fusion of neural folds of the wild type and *Grhl3^+/cre-nZ^* background (A,B,D–G). In the *Grhl3^cre-nZ/cre-nZ^* embryo, they are ectopically induced in the neural folds at the forebrain level (arrowheads in Dʹ) but not at the caudal level (C, arrows in Eʹ). Sectional views of trunk and caudal levels indicate that GRHL2-negative cells appear to be present in the vicinity of the neural folds in the *Grhl3^+/cre-nZ^* and *Grhl3^cre-nZ/cre-nZ^* at the caudal level (G,Gʹ) whereas at the trunk level, GRHL2-negative cells are not observed in the neural folds in spite of *Grhl3*–deficiency (F,Fʹ). (H–Nʹ) Morphological and molecular markers analyses of the wild type (H–N) and *CAG-Grhl2* embryos (Hʹ–Nʹ)*.* Morphological features of the gross appearances (H,Hʹ) and frontal sections (I–Jʹ) in the wild type (H–J) and *CAG-Grhl2* (Hʹ–Jʹ) embryos at E9.5. (K–Lʹ) Whole-mount *in situ* hybridization in wild-type (K,L) and *CAG-Grhl2* (Kʹ,Lʹ) using *Tfap2c* (K,Kʹ) and *Foxi2* (L,Lʹ) probes at E8.5. These SE markers were enhanced in *CAG-Grhl2* embryos (Kʹ,Lʹ). (M–Nʹ) Immunohistochemical staining in wild-type (M,N) and *CAG-Grhl2* (Mʹ,Nʹ) embryos using TROMAI antibody at E8.5. TROMAI expression (marked by magenta) was ectopically induced in the NE region in the *CAG-Grhl2* embryos (Mʹ,Nʹ). Scale bars: 100 μm in A–Eʹ, M–Nʹ; 50 μm in F–Gʹ.

**
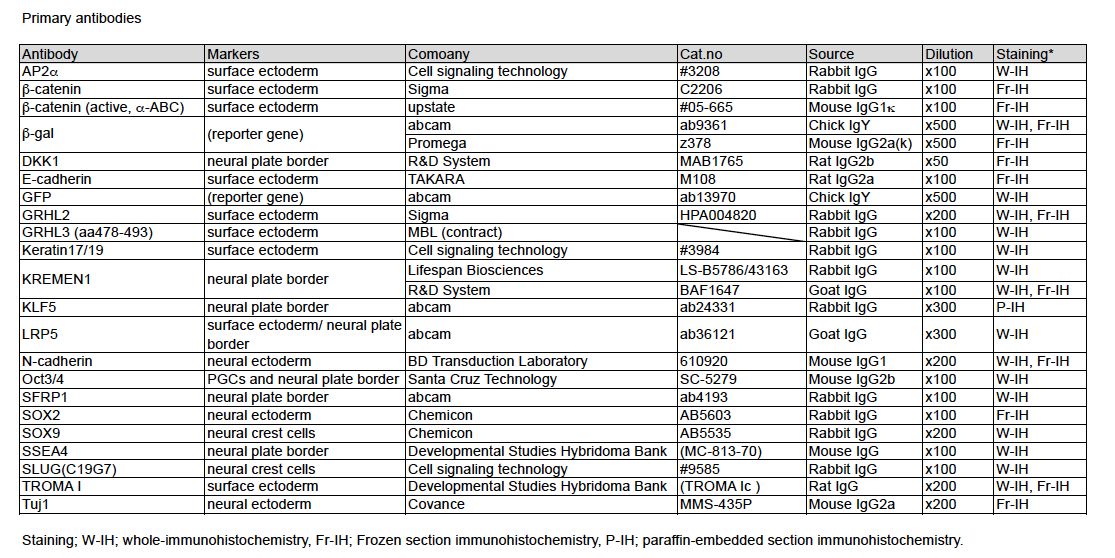
**

**Fig. S9. (related to Figs. 1–8) A list of primary antibodies used in this study.**
